# Force-driven single-molecule transformations through tip-mediated stabilization of transition states

**DOI:** 10.1038/s41467-026-78541-6

**Published:** 2026-10-23

**Authors:** Kota Iwata, Ikutaro Hamada, Ranmaru Kuno, Taku Enomoto, Ryu Kuroki, Hidehiro Sakurai, Yoshiaki Sugimoto

**Affiliations:** 1 Department of Advanced Materials Science, Graduate School of Frontier Sciences, University of Tokyo, Kashiwa, Japan; 2 Department of Precision Engineering, Graduate School of Engineering, The University of Osaka, Suita, Japan; 3 Research Center for Precision Engineering, Graduate School of Engineering, The University of Osaka, Suita, Japan; 4 Division of Applied Chemistry, Graduate School of Engineering, The University of Osaka, Suita, Japan; 5 Innovative Catalysis Science Division, Institute for Open and Transdisciplinary Research Initiatives (ICS-OTRI), The University of Osaka, Suita, Japan; 6 Department of Chemistry, Faculty of Sciences, Chulalongkorn University, Bangkok, Thailand

**Keywords:** Scanning probe microscopy, Atomic force microscopy, Chemical physics

## Abstract

Mechanochemistry uses mechanical forces to drive chemical transformations, offering an energy-efficient alternative to traditional thermal, optical, or electrical reaction pathways. At the single-molecule scale, controlling structural changes with mechanical stimuli is fundamental for developing nanoscale devices and understanding chemical reactivity. In conventional single-molecule reactions driven by light or electrical currents, energy is supplied through one-way excitation processes, requiring the single-event energy input to exceed the activation energy barrier. Here we show that mechanically driven single-molecule reactions can bypass this limitation, proceeding with a net energy cost below the activation energy. By using atomic force microscopy to induce the structural inversion of individual, bowl-shaped sumanene molecules on a gold surface, we measure the mechanical energy spent during the transformation. The net energy cost is significantly lower than the theoretical activation energy. This sub-barrier reaction occurs because attractive van der Waals forces from the mesoscopic tip body stabilize the transition state more effectively than the final state. Our findings demonstrate a distinct energetic mechanism in mechanochemistry, highlighting alternative strategies for energy-efficient molecular manipulation.

## Abstract (web summary)

Conventional chemical reactions require energy inputs exceeding activation barriers. Here, the authors demonstrate that mechanical force can drive single-molecule reactions with sub-barrier energy costs using atomic force microscopy.

## Introduction

Chemical reactions are traditionally driven by thermal, electrical, or optical energy inputs. Alternatively, mechanical stimuli—such as compression, tension, and shear—can initiate chemical transformations, a field known as mechanochemistry^1–3^. Mechanochemical processes are garnering attention as energy-efficient and environmentally benign alternatives because they often proceed without external heating or solvents^4,5^. Reported applications range from solid–solid reactions^2^ and polymorphic phase transitions^6^ to the catalysis of complexation and organic synthesis^4,7^. To date, these processes have primarily been characterized using ensemble-averaging techniques, including mass spectrometry^8^, infrared and Raman spectroscopy^2,7,9^, solid-state nuclear magnetic resonance^2^, and X-ray diffraction^9^. However, these bulk methods obscure the microscopic details of how local mechanical stimuli translate into bond cleavage or formation, leaving single-molecule reaction mechanisms largely unexplored. Triggering and observing reactions at the single-molecule level is therefore essential to elucidate the precise relationship between the applied force, energy input, and the resulting chemical structural changes.

Scanning probe microscopy has enabled the investigation of diverse chemical reactions at the single-molecule level. Reactions of adsorbed molecules have been triggered by thermal annealing^10,11^, light irradiation^12,13^, and electron injection via scanning tunneling microscopy (STM)^14^. In conventional single-molecule reactions triggered by inelastic electron tunneling or photon absorption, the external energy is supplied in a one-way process. After the reactions, the input energy cannot be recovered, with the excess energy being dissipated into the substrate as heat. Consequently, the total energy consumption in these excitation processes inevitably exceeds the activation energy.

Atomic force microscopy (AFM) using CO-functionalized tips offers the spatial resolution required to visualize internal chemical bond structures^15^. Consequently, this technique has been widely adopted to identify the reaction products generated by these conventional stimuli^16–19^. Beyond imaging, AFM is ideally suited to advance single-molecule mechanochemistry because it can directly quantify the forces and potential energies governing tip–molecule interactions. However, despite this potential, studies explicitly investigating the structural response of organic molecules to controlled mechanical stimuli remain limited^20–25^.

In this study, we demonstrate that for mechanically induced reactions, the net energy consumption can be lower than the activation energy. We investigate the structural inversion of sumanene, a bowl-shaped molecule, driven by repulsion from an AFM tip. By comparing the net energy consumption derived from force spectroscopy with the activation energy obtained using density functional theory (DFT) calculations, we verify that the reaction proceeds with sub-barrier energy consumption.

## Results and discussion

### Theoretical framework

**Fig. 1:**
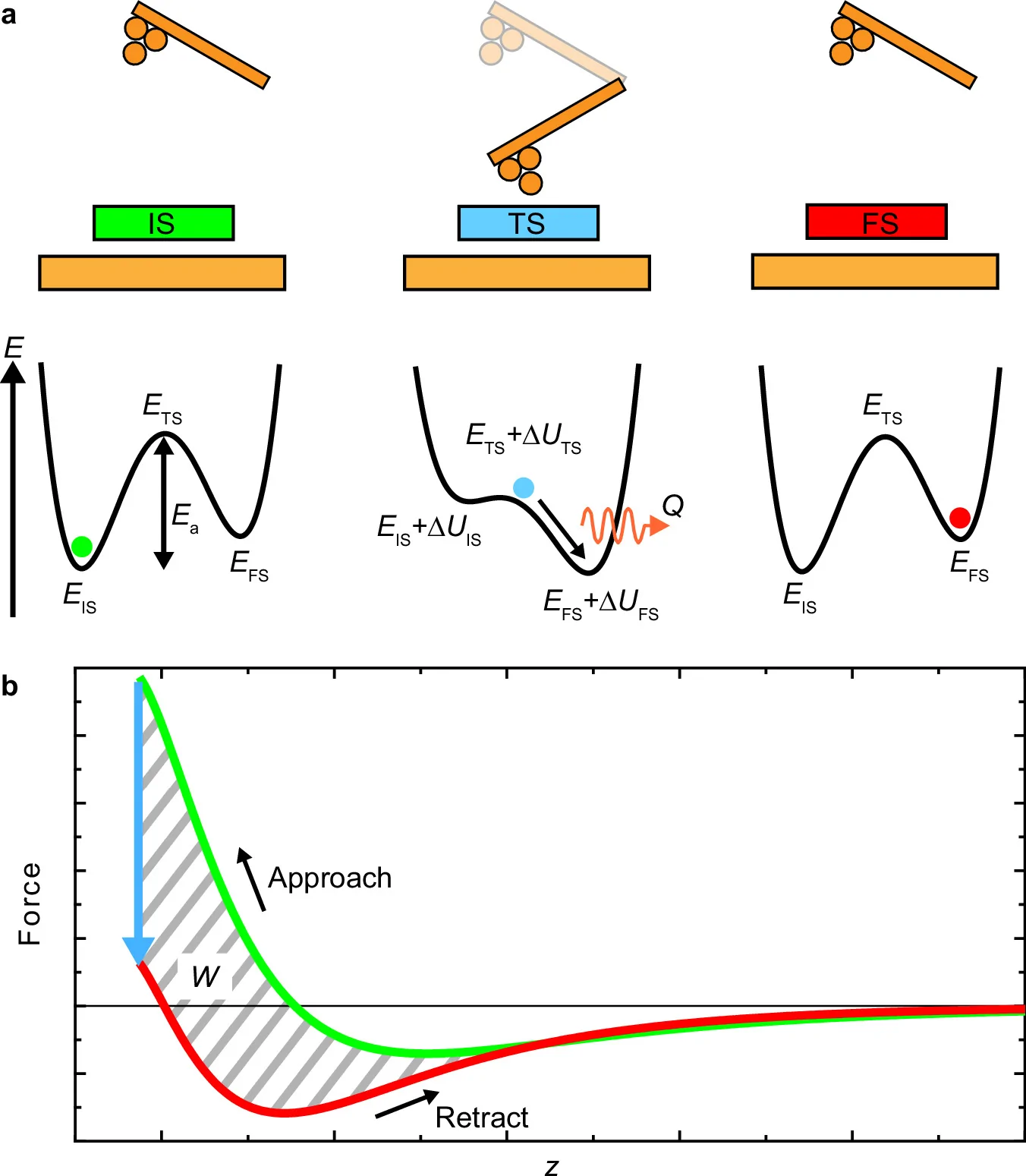
Concept of force-induced reaction in a single molecule. **a** Schematic illustration of potential energy landscapes during the reaction over one cycle of cantilever oscillation. (IS) When the tip is far from reactant, the system resides in the initial state (*E*_IS_), separated from the final state (*E*_FS_) by the transition state (*E*_TS_), which defines an activation energy ${E}_{{{\rm{a}}}}\left(={E}_{{{\rm{TS}}}}-{E}_{{{\rm{IS}}}}\right)$. (TS) Tip approach introduces a potential perturbation (Δ*U*), distorting the landscape to eliminate the barrier. The system relaxes to the new minimum, releasing heat *Q*. (FS) Upon tip retraction, the molecule remains in the final state. **b** Schematic force curves of the reaction. The plots follow the sequences: (IS) the tip approaches the reactant from a distance (light green); (TS) at the point of closest approach, the system reaches the transition state and the reaction occurs, indicated by abrupt change in force (light blue); and (FS) the tip retracts (red). Shaded area corresponds to the energy input (*W*).

We first theoretically illustrate how a chemical reaction induced by tip–molecule mechanical interactions can proceed with a net energy cost smaller than the activation energy. Consider the energy exchange during a mechanically induced single-molecule reaction. Letting *E*_IS_, *E*_TS_, and *E*_FS_ be the energies of the reactant (initial state, IS), transition state (TS), and product (final state, FS), respectively, the activation energy is defined as ${E}_{{{\rm{a}}}}={E}_{{{\rm{TS}}}}-{E}_{{{\rm{IS}}}}$ (Fig. 1a). The reaction process driven by the tip is modeled in three steps: (IS) the tip approaches the reactant from a distance; (TS) at the point of closest approach, the system reaches the transition state and the reaction occurs; and (FS) the tip retracts. We assume that the sample is gradually brought closer to the oscillating tip. If the distance between the sample and the tip at the lowest turnaround point of the oscillation is too large, the reaction does not occur. When the reaction is observed at a specific distance, it must occur at the exact moment when the tip reaches its closest approach to the sample during the oscillation. The reaction proceeds deterministically with respect to the tip position at a sufficiently low temperature. This instantaneous moment of reaction corresponds to TS configuration sketched in Fig. 1a.

Let *W* be the net energy input to the molecular system that interacts with the tip in this process. Assuming heat *Q* is dissipated only during the instantaneous relaxation from the transition state to the final state (an irreversible process), energy conservation gives $W=\left({E}_{{{\rm{FS}}}}-{E}_{{{\rm{IS}}}}\right)+Q$. If the tip is attached to an oscillator (cantilever), *W* corresponds to the mechanical energy lost by the oscillator, which equals the area enclosed by the forward and backward force curves (hysteresis) shown schematically in Fig. 1b.

To compare the energy cost *W* and the activation energy *E*_a_, we consider the energetics at the point of closest approach (TS). Here, the interaction with the tip (Δ*U*) modifies the intrinsic system energies to ${E}_{{{\rm{IS}}}}+{\Delta U}_{{{\rm{IS}}}}$, ${E}_{{{\rm{TS}}}}+{\Delta U}_{{{\rm{TS}}}}$, and ${E}_{{{\rm{FS}}}}+{\Delta U}_{{{\rm{FS}}}}$. The heat released is $Q=\left({E}_{{{\rm{TS}}}}+{\Delta U}_{{{\rm{TS}}}}\right)-\left({E}_{{{\rm{FS}}}}+{\Delta U}_{{{\rm{FS}}}}\right)$. Substituting this into the conservation equation yields $W-{E}_{{{\rm{a}}}}={\Delta U}_{{{\rm{TS}}}}-{\Delta U}_{{{\rm{FS}}}}$.

If only attractive (repulsive) forces act during the reaction, typically ${\Delta U}_{{{\rm{FS}}}} < {\Delta U}_{{{\rm{TS}}}} < 0$ (${0 < \Delta U}_{{{\rm{FS}}}} < {\Delta U}_{{{\rm{TS}}}}$), requiring ${E}_{{{\rm{a}}}} < W$. However, if attractive interactions coexist with repulsion such that ${\Delta U}_{{{\rm{TS}}}} < {\Delta U}_{{{\rm{FS}}}}$ (i.e., the transition state is more stabilized by the tip than the final state), the reaction can proceed with $W < {E}_{{{\rm{a}}}}$. This conclusion highlights a feature of mechanical manipulation: unlike electron or photon excitation where excess energy is lost, mechanical interactions allow energy saving (detailed simulation provided in Supplementary Note 6).

### Experimental verification with sumanene

**Fig. 2:**
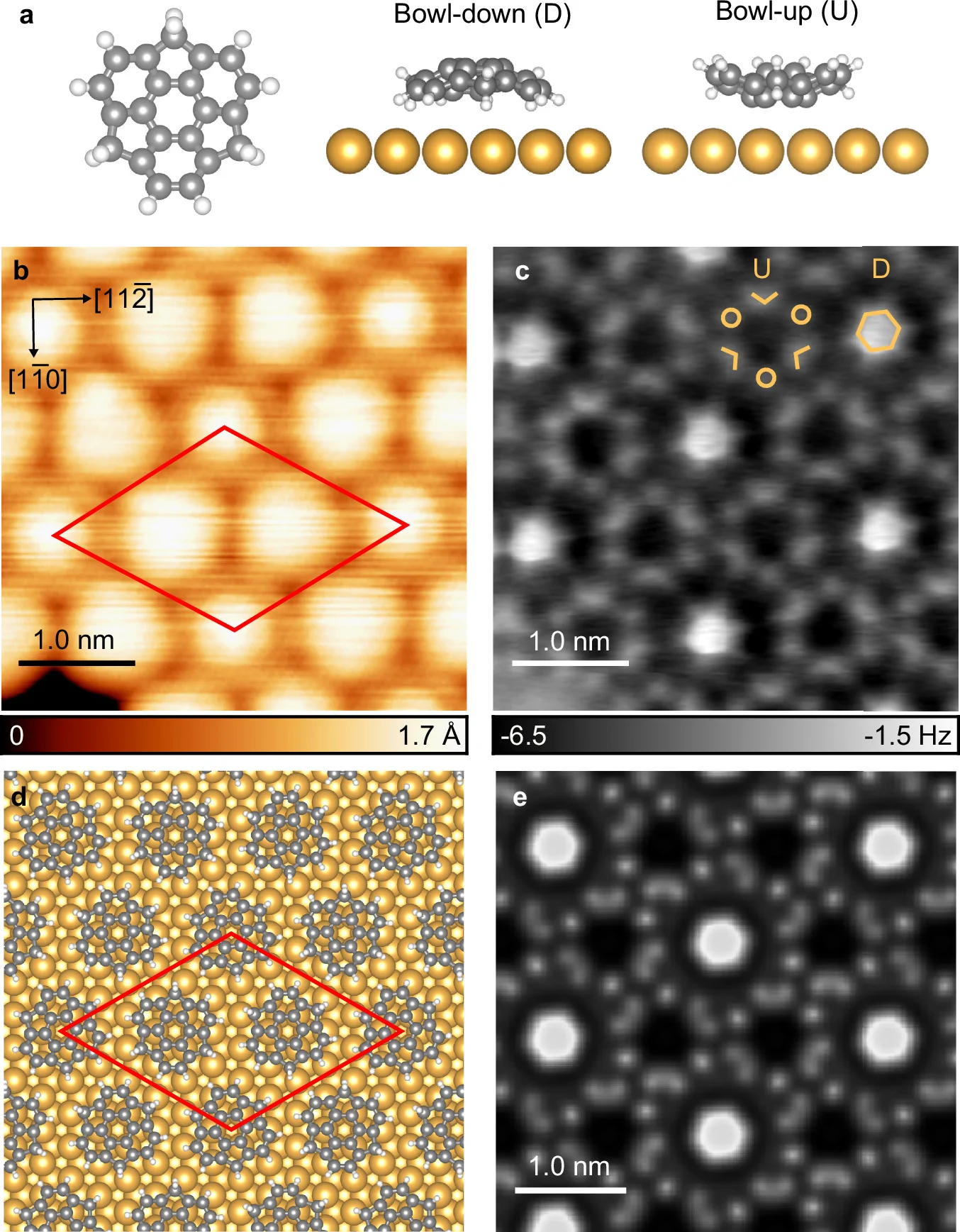
Structure and imaging of sumanene on Au(111). **a** Top and side views of the sumanene molecule. **b** STM image of a self-assembled sumanene monolayer on Au(111) acquired with a CO-terminated tip (*V*_S_ = 50 mV, *I*_t_ = 10 pA). The red rhombus indicates the unit cell. **c** Constant-height AFM image of the same area as in (**b**). Two distinct molecular appearances are denoted as U (bowl-up) and D (bowl-down). Orange lines schematically highlight characteristic features. **d** Energetically most stable adsorption structure obtained by DFT calculations. Gray, white, and yellow spheres represent carbon, hydrogen, and gold atoms, respectively. **e** Simulated AFM image corresponding to the structure in (**d**) using the probe particle model.

To verify the $W < {E}_{{{\rm{a}}}}$ hypothesis, we selected sumanene molecules adsorbed on an Au(111) substrate. Sumanene is a “buckybowl” (a fragment of fullerene)^26^ and is ideal for this study because its steric structure, “bowl-down” (D) and “bowl-up” (U), allows for inversion via repulsion with the tip (Fig. 2a). Our DFT calculations estimate the inversion barrier for a gas-phase sumanene molecule to be 0.91 eV, consistent with previous reports (≈0.82 eV)^27,28^. When adsorbed, the reaction coordinate for inversion aligns with the surface normal, making it accessible to the AFM tip.

Figure 2b shows a typical STM image of sumanene monolayer on Au(111). The film exhibits a periodic array of two distinct bright features with a lattice constant of 1.88 nm. The crystallographic orientation of the Au(111) substrate was determined based on the herringbone reconstruction (Supplementary Fig. 1). From this alignment, we identified the film as a commensurate (6 × 6) superstructure.

To determine the molecular orientation and rotation, which are difficult to resolve by STM alone, we performed high-resolution AFM imaging with a CO-terminated tip (Fig. 2c). Due to the curved structure of sumanene, the AFM images reflect the molecular moieties protruding toward the vacuum. We identify the two features as the D and U orientations, visualizing the central six-membered ring and the molecular rim, respectively. Specifically, molecule D appears as a distorted hexagon due to bond-order effects within the central ring^29^, while molecule U exhibits a circular arrangement of V-shapes and spots, reflecting the arrangement of its constituent five- and six-membered rings. The unit cell consists of a “2U1D” configuration, which contains two U and one D sumanene molecules. These detailed intra-molecular features allow us to determine the precise azimuthal rotation of the molecules. We observed that the molecular symmetry axes are rotated relative to the superlattice vectors, revealing that the sumanene monolayer possesses chirality.

We determined the adsorption structure using DFT calculations with a van der Waals (vdW)-inclusive exchange-correlation functional^30^. For single molecules, the U configuration at the face-centered cubic hollow site is the most stable. For the monolayer, a systematic search identified the 2U1D structure (Fig. 2d) as the energetic minimum (Supplementary Figs. 2, 3 and Supplementary Note 3). AFM simulations based on the probe particle model^31^ using this structure (Fig. 2e) are consistent with experimental images.

### Tip-induced molecular inversion

**Fig. 3:**
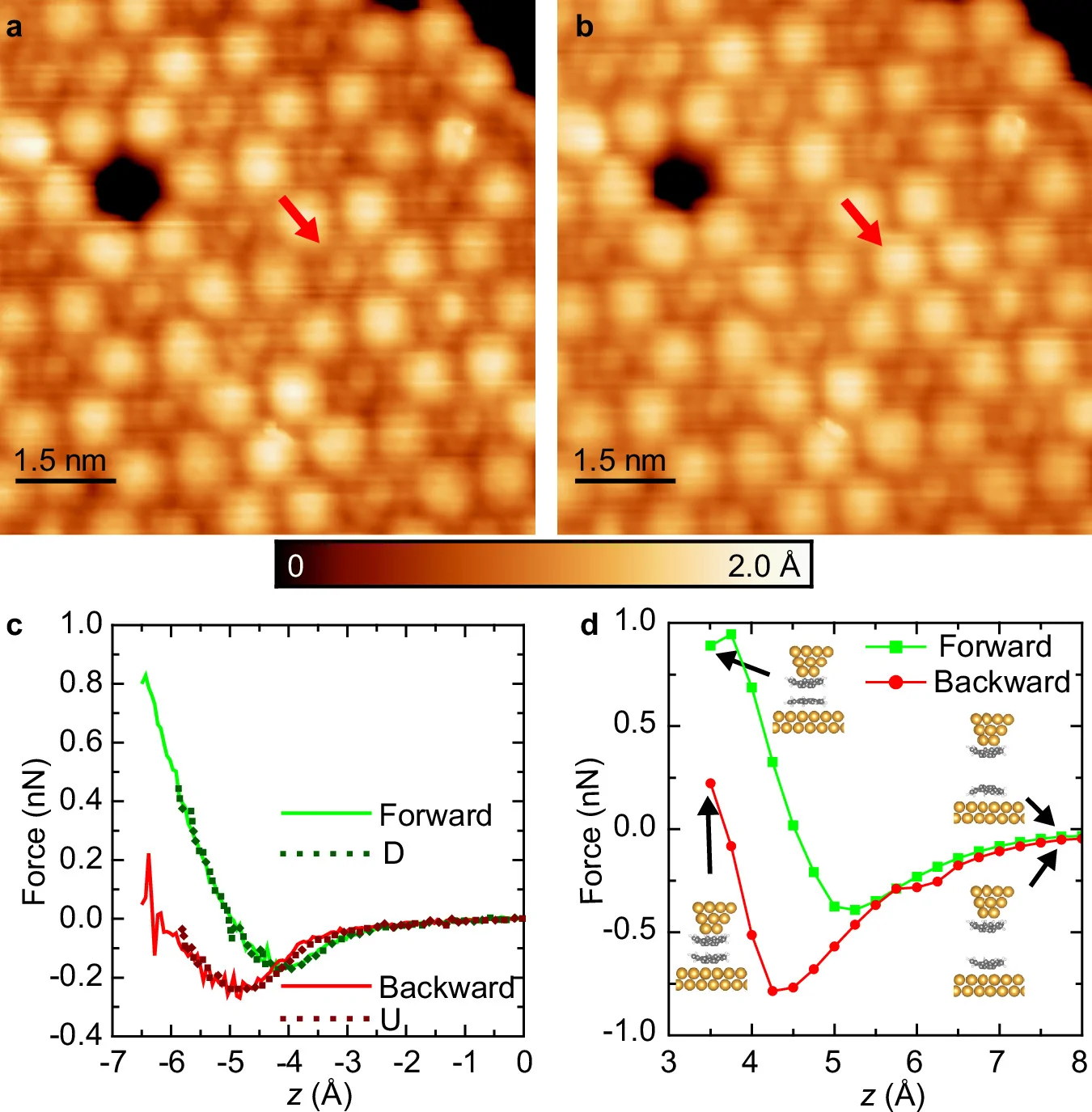
Mechanical inversion of sumanene. **a**, **b** STM images before and after the manipulation, respectively (*V*_S_ = 50 mV, *I*_t_ = 10 pA). The red arrows indicate the targeted sumanene molecule. **c** Force curves recorded during the inversion. Solid light green and red curves represent the approach and retraction paths during a successful inversion, respectively. Dotted curves indicate reference measurements on bowl-down (green) and bowl-up (brown) molecules where the tip approach was stopped before triggering the inversion. **d** Simulated force curves for the inversion event using a sumanene-terminated Au_19_ tip. The inset shows the snapshots of the system during the inversion process. Source data are provided as a Source Data file.

We experimentally demonstrate that the tip–molecule mechanical interaction can induce structural inversion. The tip was positioned over a D molecule (Fig. 3a) and approached toward the molecule. At a threshold distance, a discontinuity (jump) appeared in the frequency shift curve, resulting in hysteresis between the forward and backward paths (Supplementary Fig. 8). Subsequent STM imaging (Fig. 3b) confirmed that the target D molecule had converted to a U appearance. Crucially, neither the inverted molecule nor the surrounding molecules exhibited rotational changes, confirming that only the targeted single-molecule inversion took place (Supplementary Fig. 4).

Figure 3c displays the short-range force curve converted from the frequency shift^32^. During approach (light green), the force evolves from attractive to repulsive. As repulsion increases, a sudden jump occurs. The tip then retracts, tracing a different force trajectory (red) due to the altered interaction. Force curves measured over D and U molecules within a distance range where inversion does not occur are also shown as dotted curves in Fig. 3c. These curves match the pre-jump (forward) and post-jump (backward) traces, respectively, confirming that the jump corresponds to the instantaneous structural inversion from bowl-down to bowl-up. The intramolecular contrast in the STM images (Fig. 3a, b) suggests the use of a molecule-functionalized tip, as we suspect a sumanene molecule was inadvertently picked up by the tip during the experiments. As discussed in Supplementary Note 9 and Supplementary Fig. 10, a bare metallic tip tends to pick up sumanene molecules from the surface.

DFT calculations of a sumanene-terminated pyramidal metal tip approaching a surface-adsorbed D molecule (Fig. 3d) qualitatively reproduce the experimental force curves. As the tip approaches (z<4 Å), repulsion flattens the bowl. At z=3.5 Å, the molecule reaches a flat transition state and instantaneously snaps to the U configuration. Similarly, simulations with two different metal tip geometries yielded results consistent with the sumanene-terminated tip, confirming that the molecule deforms under repulsion and inverts upon reaching the transition state (Supplementary Fig. 5, Supplementary Movies 1, 2). Unlike molecular manipulation by attractive forces^22,23^, this is a mechanical manipulation that does not require the chemical reactivity of the tip.

### Energy cost and activation energy analysis

**Fig. 4:**
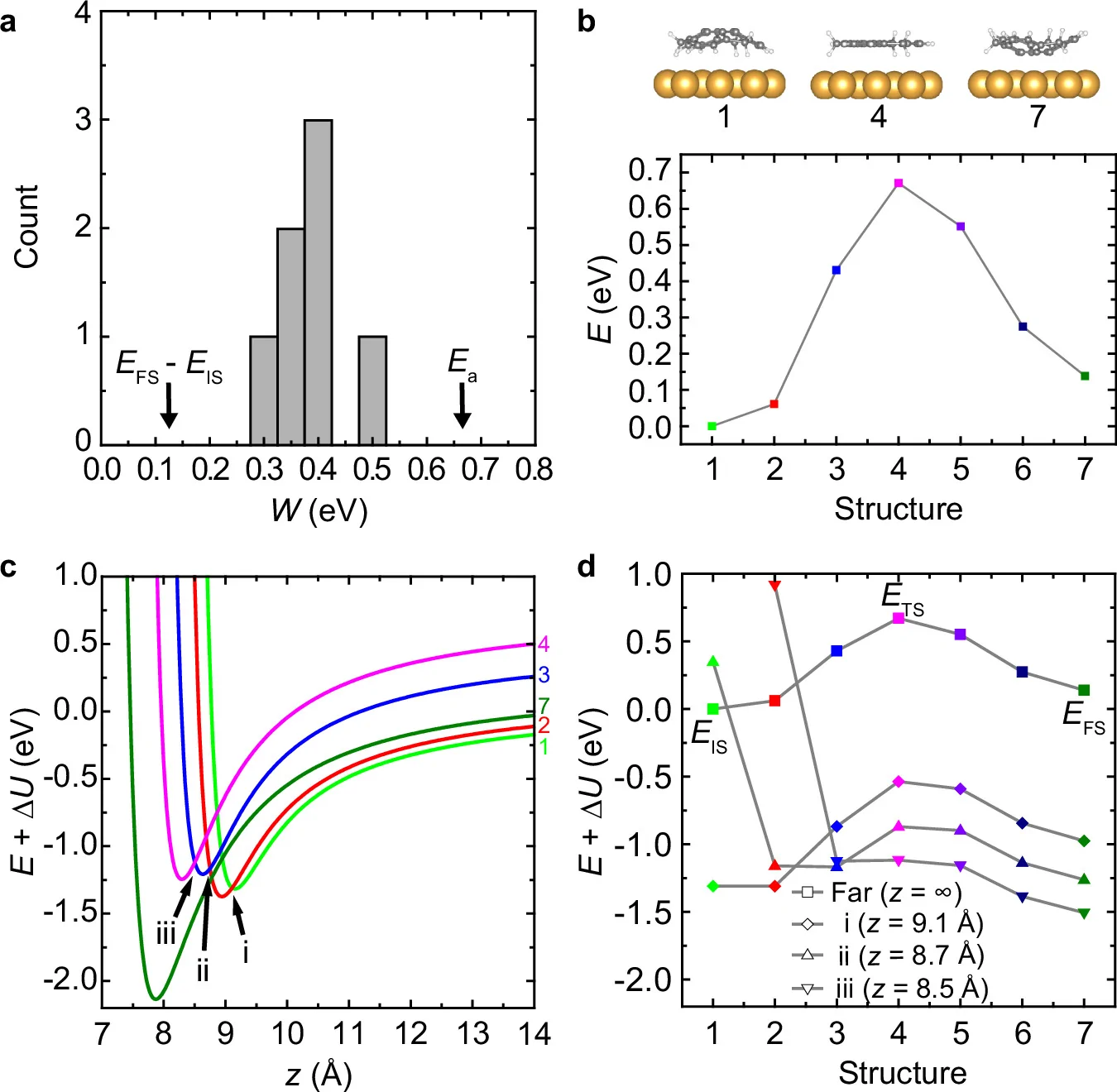
Energetics of the inversion. **a** Histogram of the measured *W* obtained through multiple manipulations using different tips. **b** Relative energy (*E*) along the reaction coordinate obtained using the DFT calculations with the NEB method. Structures 1 (light green), 4 (pink), and 7 (green) correspond to the initial, transition, and final states, respectively, while 2 (red), 3 (blue), 5 (purple), and 6 (navy) represent intermediate structures. **c** Simulated total energy evolution of each state in the presence of the tip as a function of tip height. The tip was modeled using a 219-atom truncated tetrahedral Au cluster. Intersections labeled i, ii, and iii correspond to the crossover points between structures 1–2, 2–3, and 3–4, respectively. **d** Evolution of the energy profile along the reaction coordinate. The data points are taken from (**b**) for far distances and from (**c**) for the distances corresponding to the intersections i, ii, and iii. Source data are provided as a Source Data file.

We now test the hypothesis $W < {E}_{{{\rm{a}}}}$. The mechanical energy lost by the cantilever is the area of the hysteresis loop in the force curves (Fig. 3c), measured here as *W* = 0.50 eV. The effect of non-conservative forces on this energy estimate is negligible (Supplementary Fig. 8). Figure 4a summarizes *W* measured across multiple inversion events with different tips.

To compare with *W*, the activation energy *E*_a_ is required. However, *E*_a_ cannot be determined experimentally because the reaction does not proceed thermally. Therefore, we theoretically calculated *E*_a_ using the nudged elastic band (NEB) method^33,34^(Fig. 4b). The inversion creates a locally strained 3U structure, making the final state higher in energy than the initial state (${E}_{{{\rm{IS}}}} < {E}_{{{\rm{FS}}}}$). The calculated *E*_a_ of 0.67 eV is consistent with theoretical values on Ag^35^ and Au^36^ surfaces. Crucially, values of *W* are consistently smaller than the calculated activation energy ($W < {E}_{{{\rm{a}}}}$). On the other hand, since $W > {E}_{{\mbox{FS}}}-{E}_{{\mbox{IS}}}$, Q>0 is satisfied, and the law of conservation of energy is not violated.

### Mechanism of energy savings

We attribute the observed energy savings ($W < {E}_{{{\rm{a}}}}$) to the interplay between short-range repulsion from the tip apex and vdW attraction from the mesoscopic “tip body.” During the approach, the cantilever gains mechanical energy as the vdW attraction performs work on the tip prior to the repulsive deformation. Because the vdW attraction is reduced during retraction after the inversion, the interaction yields a net energy gain over a single oscillation cycle. This gain partially compensates for the energy consumed by the repulsive deformation, effectively reducing the net energy cost. In other words, the transition state is more stabilized by the tip interaction than the final state (${\Delta U}_{{{\rm{TS}}}} < {\Delta U}_{{{\rm{FS}}}}$).

To validate this interpretation, we evaluated the total system energy (E+ΔU) as the sum of the intrinsic energy (*E*) of each state and the energy shift induced by a Lennard-Jones potential (ΔU) that accounts for the massive tip body as well as the tip apex (Fig. 4c, d and Supplementary Figs. 6), as the DFT calculations are limited to microscopic tip models that cannot capture the substantial vdW attraction, leading to ${E}_{{{\rm{a}}}} < W$. As the tip approaches, the system undergoes barrier-less transitions at intersections i (structure 1→2) and ii (structure 2→3), leading to the final inversion from the transition state (structure 4) to the product (structure 7) at intersection iii. This model yields ${\Delta U}_{{{\rm{TS}}}} < {\Delta U}_{{{\rm{FS}}}}$ (${\Delta U}_{{{\rm{TS}}}}=$−1.80 eV vs. ${\Delta U}_{{{\rm{FS}}}}=$−1.65 eV), reproducing the $W < {E}_{{{\rm{a}}}}$ condition. The Au tip capped with the sumanene also reproduced the $W < {E}_{{{\rm{a}}}}$ condition although the value of W differed slightly (Supplementary Fig. 7). The variance in measured *W* (Fig. 4a) likely reflects the geometric diversity of the tips.

We demonstrated the single-molecule structural manipulation of sumanene using AFM. By applying repulsive mechanical force to a bowl-down molecule, we induced an inversion to the bowl-up state. Force spectroscopy revealed that the net mechanical energy cost was significantly lower than the activation energy of the reaction. This stands in contrast to conventional chemical reactions, where the supplied energy must exceed the activation barrier. In this mechanically induced process, the continuous interaction with the tip facilitates energy savings through vdW attraction, enabling the reaction to proceed with sub-barrier energy consumption. Our findings suggest that mechanochemical reactions offer not only alternative reaction pathways but also an energetic advantage.

## Methods

### STM/AFM measurements

All experiments were performed using an Omicron low-temperature AFM/STM system operating under ultrahigh vacuum (UHV) conditions at 4.8 K. We utilized a qPlus sensor equipped with a tungsten tip. The typical sensor parameters were a spring constant $k\approx 2000\,{{\rm{N}}}{{{\rm{m}}}}^{-1}$, a resonant frequency ${f}_{0}\approx 18.5\,{{\rm{kHz}}}$, and a quality factor $Q\approx {\mathrm{10,000}}$. During AFM operation, the oscillation amplitude was maintained at 100 pm. We employ a highly accurate calibration method of the oscillation amplitude based on the displacement of the sample stage when changing the oscillation amplitude under tunneling current feedback control, with an error of no more than 10%. The bias voltage *V*_S_ refers to the sample bias with respect to the tip. STM measurements were conducted in constant-current mode. High-resolution AFM imaging was performed in constant-height mode with ${V}_{{{\rm{S}}}}=0\,{{\rm{V}}}$. Force spectroscopy measurements were also acquired at zero bias (${V}_{{{\rm{S}}}}=0\,{{\rm{V}}}$).

The Au(111) substrate was cleaned by repeated cycles of Ar^+^ sputtering and annealing. Sumanene molecules were thermally evaporated onto the clean Au(111) substrate held at room temperature. After cooling the sample, a trace amount of CO gas was dosed onto the substrate at $\approx \,$ 8 K. For the high-resolution imaging, the CO-terminated tip was prepared by picking up a single CO molecule from the bare Au(111) surface.

### DFT calculations

All DFT calculations were performed using the STATE^37^ code. Electron-ion interactions were described using pseudopotentials: H 1 *s*, C 2*p*, and Au 3 *d* channels were described with the Vanderbilt ultrasoft pseudopotential scheme^38,39^, whereas other channels were described with the Troullier-Martins norm-conserving pseudopotential scheme^40^. Wave functions and charge density were expanded in terms of a plane-wave basis set with the kinetic energy cutoffs of 36 Ry and 400 Ry, respectively. The exchange-correlation functional was described using rev-vdW-DF2^30^, a variant of the van der Waals density functional^41^, which has been shown to describe adsorption of organic molecules on metal surfaces accurately^42^. The Au(111) substrate was modeled using a slab composed of three Au layers with a vacuum equivalent to an eight Au layer thickness, which was periodic in surface parallel directions. To investigate their adsorption states, the sumanene molecules were placed on one side of an Au(111) surface with a (6 × 6) periodicity with a non-shifted 2 × 2 k-point set, and spurious electrostatic interaction with neighboring slabs was eliminated using the effective screening medium (ESM) method^43,44^. The atoms in the bottommost layer of the slab were fixed to their respective bulk Au positions, while other atoms were fully relaxed until the forces acting on them were smaller than 2.5 × 10^−2^ eV Å^−1^ (1 × 10^−3^ Hartree Bohr^−1^). To investigate the tip-induced inversion of single sumanene molecule, we used a simulation cell with a thicker vacuum equivalent to a fifteen Au layer thickness and introduced a pyramidal Au(111) tip composed of twenty Au atoms (Au_20_), that with missing apex Au atom (Au_19_), and Au_19_ tip functionalized with a sumanene molecule in the bowl-down configuration. The top two atomic layers of the model tips were fixed at their respective bulk Au positions, while other degrees of freedom were fully relaxed. The force curves were calculated by differentiating the interaction energy curves with respect to the tip-sample distance in the surface normal direction. When the model tips were introduced, the ESM method was not used, to reduce the computational cost associated with the increased vacuum thickness. To calculate the activation energy for the bowl inversion of a single sumanene molecule in the absence of the tip, the NEB method^33^ with the climbing image^34^ was used. To investigate the effect of the interaction between the molecules in the periodic images in the lateral directions on the activation energy, Au(111) (6 × 6), (6 × 6√3), and (12 × 12) supercells with a vacuum equivalent to eight Au layers were also used. We obtained the activation energies of 0.744, 0.694, and 0.671 eV for the three supercells, respectively. By extrapolation, we estimated a value of 0.61 eV for the isolated limit. This estimate quantifies the error associated with the use of the periodic boundary conditions.

### Reporting summary

Further information on research design is available in the Nature Portfolio Reporting Summary linked to this article.

## Supplementary information

Supplementary Information

Description of Additional Supplementary File

Supplementary Movie 1

Supplementary Movie 2

Reporting Summary

Transparent Peer Review file

## Source data

Source data

## Data Availability

The data that support the findings of this study, including the DFT data, are available at Zenodo^45^ and from the corresponding authors upon request. Source data are provided with this paper.
