# Supplementary material for: Force-driven single-molecule transformations through tip-mediated stabilization of transition states
Source: Nat Commun. 2026 Oct 23;17:10868. doi: 10.1038/s41467-026-78541-6 (PMC13694703; doi:10.1038/s41467-026-78541-6)
Supplement: Supplementary file 1 [file 41467_2026_78541_MOESM1_ESM.pdf]

Supplementary Information for

Sub-barrier energy cost in force-driven single-molecule structural transformation

Kota Iwata, Ikutaro Hamada, Ranmaru Kuno, Taku Enomoto, Ryu Kuroki, Hidehiro Sakurai, Yoshiaki Sugimoto

Corresponding author: ysugimoto@k.u-tokyo.ac.jp

The PDF file includes:

Supplementary Note 1 to 13
Supplementary Figures 1 to 11
Supplementary References

Supplementary Note1. General condition for $W < E_a$ in tip-induced reactions

Here, we generalize the condition under which the energy cost W becomes smaller than the activation energy E_a, considering cases where the reaction occurs before the system reaches the original transition state (TS). When the tip approaches, the potential energy surface is modified. If the activation barrier vanishes due to the tip interaction before the molecule reaches the original TS geometry, the system will "jump" to the final state from an intermediate configuration. Let the energy and the tip–molecule interaction energy at this jump point be E'_{TS} and $\Delta U'_{\mathrm{TS}}$, respectively. The heat dissipated during the jump is given by:

$$Q = (E'_{\mathrm{TS}} + \Delta U'_{\mathrm{TS}}) - (E_{\mathrm{FS}} + \Delta U_{\mathrm{FS}}) \quad (1)$$

Substituting this into the energy conservation equation $W = (E_{\mathrm{FS}} - E_{\mathrm{IS}}) + Q$ yields:

$$W = (E_{\mathrm{FS}} - E_{\mathrm{IS}}) + (E'_{\mathrm{TS}} + \Delta U'_{\mathrm{TS}}) - (E_{\mathrm{FS}} + \Delta U_{\mathrm{FS}})$$
$$= E'_{\mathrm{TS}} - E_{\mathrm{IS}} + \Delta U'_{\mathrm{TS}} - \Delta U_{\mathrm{FS}} \quad (2)$$

Subtracting the intrinsic activation energy ($E_a = E_{\mathrm{TS}} - E_{\mathrm{IS}}$) from both sides, we obtain:

$$W - E_a = (E'_{\mathrm{TS}} - E_{\mathrm{TS}}) + (\Delta U'_{\mathrm{TS}} - \Delta U_{\mathrm{FS}}) \quad (3)$$

Since the jump occurs before the original TS is reached (i.e., at a lower energy configuration along the reaction coordinate), $E'_{\mathrm{TS}} - E_{\mathrm{TS}} < 0$. Therefore, if $\Delta U'_{\mathrm{TS}} - \Delta U_{\mathrm{FS}} < 0$ (meaning the transition state–or the pre-transition jump point–is more stabilized by the tip than the final state), the condition $W < E_a$ is satisfied.

Supplementary Note 2. Large-scale STM imaging and comparison with previous reports

Supplementary Figure 1 displays large-scale STM images of the sumanene adlayer acquired at 5 K (Supplementary Figure 1a) and 80 K (Supplementary Figure 1b). Upon depositing a sub-monolayer of sumanene, the molecules form islands, leaving bare Au(111) regions at both temperatures. Inside the islands, the molecules form the "2U1D" structure described in the main text. The crystallographic orientation of the Au(111) substrate was determined from the herringbone reconstruction pattern (indicated by arrows in Supplementary Figure 1).

Previous STM and DFT studies on sumanene adsorbed on Au(111) reported that all molecules adopt the Bowl-up (U) configuration (*1*). This discrepancy likely arises from differences in sample preparation. The previous study prepared samples by immersing the Au substrate in a sumanene solution, which may have resulted in a multilayer film or a specific solution-mediated structure. In contrast, our study employed vacuum deposition, resulting in the formation of a monolayer. Notably, our observed structure is consistent with reports of vacuum-deposited sumanene on Ag(111), where an analogous 2U1D structure within the molecular islands was confirmed by STM and DFT (*2*).

Supplementary Note 3. DFT structure determination

In this study, we systematically investigated the adsorption states of a sumanene molecule and monolayer using DFT calculations as follows:

For the single sumanene molecule, we considered the four high-symmetry adsorption sites on Au(111) (Supplementary Figure 2a) and placed a sumanene molecule in either a bowl-up or bowl-down configurations with the center of the central hexagon at the adsorption sites. Nonequivalent molecular orientations, namely 30° and 0°, in which the molecular mirror plane aligns with the *x* and *y* axes, respectively, were also considered. The stability of the molecule in different adsorption states was examined by calculating the adsorption energy, defined as

$$E_{\mathrm{ads}} = \frac{[E_{\mathrm{tot}}(\mathrm{SUM/S}) - n_{\mathrm{SUM}} E_{\mathrm{tot}}(\mathrm{SUM}) - E_{\mathrm{tot}}(\mathrm{S})]}{n_{\mathrm{SUM}}}, \quad (4)$$

where $E_{tot}(SUM/S)$, $E_{tot}(SUM)$, and $E_{tot}(S)$ are total energies of the adsorption system, the sumanene molecule in the gas-phase, and the Au(111) substrate, respectively, and n_{SUM} is the number of sumanene molecules in the surface unit cell. Supplementary Figure 2b shows a single bowl-up sumanene molecule in the 30° orientation at the face-centered cubic (FCC) hollow site, which is energetically most stable among all the adsorption states considered in this study. Supplementary Figure 2c displays bowl-up molecule in the 0° orientation at the hexagonal closed-pack (HCP) hollow site, which is the most stable configuration among the sumanene molecules in this specific orientation (0°). E_{ads} calculated for the bowl-up and bowl-down single sumanene molecules at high symmetry sites in 30° and 0° orientations are shown in Supplementary Figures 2d and 2e, respectively.

For the sumanene monolayer, we first placed the three sumanene molecules in the 30° orientation on Au(111) (6×6). We considered all possible combinations of the bowl-up and bowl-down configurations at the FCC sites, namely, n_UUn_DD, where n_U and n_D indicate the number of sumanene molecules in the bowl-up and the bowl-down configurations, respectively. We found that 2U1D is the most stable monolayer structure, which is consistent with the one suggested based on the AFM observation. We then investigated the mutual orientations of the molecules within the 2U1D by manually rotating one bowl-up and one bowl-down molecule (labeled U1 and D1 in Supplementary Figure 3, respectively) and then fully relaxing the geometric structure. We found that 2U1D with U1 and D1 rotated by approximately 60° and 10°, respectively, is the most stable structure (Supplementary Figure 3a) with E_{ads} of −1.667 eV/molecule. We further introduced small random rotations to each molecule within 2U1D to generate different initial structures. After optimization of the structures, we confirmed that the obtained 2U1D structure was the most stable. Finally, we investigated the most stable adsorption site by examining other adsorption sites, namely, the top, HCP, and bridge sites. We found that E_{ads} values for the top, HCP, and bridge sites are −1.514, −1.635, and −1.633 eV/molecule, respectively. We confirmed that FCC is the most stable site, although it is energetically competitive with HCP and bridge sites. The AFM simulation (*3*) of this optimized structure produces the image shown in Supplementary Figure 3b, which agrees well with the experimental AFM image (Supplementary Figure 3c).

Supplementary Note 4. Structural identification of the inverted product

We confirmed the tip-induced inversion using high-resolution AFM imaging. Supplementary Figure 4a indicates three D molecules (light blue circles) targeted for manipulation. Similar to the procedure described in the main text, approaching the tip over the target D molecules resulted in frequency shift jumps in the repulsive regime. Subsequent STM imaging (Supplementary Figure 4b) revealed a change in the appearance of these molecules. To determine their structure, we performed constant-height AFM imaging. As shown in Supplementary Figure 4c, the manipulated molecules (light blue circles) exhibit a contrast pattern identical to native Bowl-up molecules (red circle), confirming the inversion from D to U.

Supplementary Figures 4d and 4e illustrate the symmetry analysis. The native U molecules (red arrows) align along two directions, while the D molecules (light green arrows) align along one direction within the domain. The symmetry axes of the inverted molecules (light blue arrows) coincide with the original axes of the D molecules (light green arrows). No azimuthal rotation was observed within the experimental accuracy of our AFM imaging. We therefore conclude that the molecule undergoes a direct vertical inversion without rotation.

Supplementary Note 5. DFT simulation of tip-induced inversion

To elucidate the mechanism of the tip-induced inversion, we performed DFT calculations using three different tip models placed above the sumanene molecule in the D configuration (D1) of the molecular layer with the Au(111) (6×6) surface unit cell. The atomic structures of these tips are depicted in the insets of Supplementary Figures 5b–d. All tip models successfully triggered the molecular inversion in the repulsive force regime.

Supplementary Figure 5a reproduces the experimental force curve from Fig. 3 (main text), characterized by weak attraction followed by repulsion and a jump. Supplementary Figures 5b and 5c show calculated force curves using an Au_{20} and an Au_{19} tip, respectively. The Au_{20} tip reproduces the approach behavior and triggers inversion. However, the calculated interaction energy curve for the retraction was not smooth enough to obtain a smooth force curve, although there were no significant apparent changes in the geometries of tip and the inverted sumanene molecule. Therefore, we do not show the calculated force curve for the retraction obtained using the Au_{20} tip. We presume this is because the Au_{20} tip, in particular, the apex Au atom is soft and very sensitive to the environment and stringent convergence thresholds for the total energy and forces are required to obtain smooth interaction energy and force curves. Supplementary Figure 5d shows the result using a sumanene-terminated Au_{19} tip. This model qualitatively reproduces the experimental features, including the attractive well and the hysteresis loop. Movies of the simulations using the Au_{19} tip and the sumanene-terminated Au_{19} tip are provided as Supplementary Movies.

We calculated W for these tip models. The Au_{19} tip yields W = 1.01 eV, the sumanene-terminated tip yields W = 0.97 eV, and the Au_{20} tip yields W = 1.01 eV. The activation energy obtained by the NEB calculations for the same unit cell is E_a = 0.75 eV. Thus, all these standard DFT models result in $E_{\mathrm{a}} < W$. These results necessitate the mesoscopic tip body model discussed in the "Mechanism of Energy Savings" section in the main text to explain the experimental $W < E_{\mathrm{a}}$.

Supplementary Note 6. Theoretical validation of the energy saving mechanism

We performed simulations to demonstrate that $W < E_{\mathrm{a}}$ is achievable when considering the mesoscopic tip body. The molecular structures corresponding to the images along the reaction coordinates (1 to 7) obtained from the NEB calculation (Fig. 4B, main text) were fixed as shown in Supplementary Figure 6a. The energy profile and activation energy were obtained using the three-layer Au(111) (12×12) slab. We then modeled the tip using a truncated tetrahedral Au cluster composed of 219 atoms. This tip was generated by stacking 10 atomic layers (with the n-th layer containing $n(n+1)/2$ atoms) and subsequently removing the single atom at the tip apex (Supplementary Figure 6a). The interaction was calculated as the sum of a Lennard-Jones (L-J) potential for all atomic pairs (Supplementary Figure 6b). The total system energy ($E + \Delta U$) was obtained by adding the L-J potential energy shift (ΔU) to the intrinsic energy (E) of each NEB state (Supplementary Figure 6c). That is, the energy at large z in Supplementary Figure 6c corresponds to the energy calculated using the NEB method.

As the tip approaches, the attractive interaction stabilizes the initial state. Further approach introduces repulsion, destabilizing the system and triggering a barrier-less transition from curve 1 to curve 2 at intersection i, and subsequently to curve 3 at intersection ii. Although these transitions appear discrete in our model due to the discretization of the reaction coordinate, the actual physical process involves a continuous and smooth deformation of the molecule. Finally, the system transitions to the transition state (structure 4) and snaps to the final state (structure 7) at intersection iii. After this transition, the potential energy curve of the final state no longer intersects with that

of the transition state; consequently, the system remains in the final state without transitioning to other states. Supplementary Figure 6d summarizes the potential landscape in the presence of the tip. While repulsion drives the molecule toward the transition state, the attractive contribution from the tip body lowers the overall energy of the system compared to the tip-free case. Crucially, at the tip height where the jump occurs, the tip is farther from the molecule in the final state than in the transition state. Thus, the stabilization is less pronounced for the final state, leading to the condition $\Delta U_{\mathrm{TS}} < \Delta U_{\mathrm{FS}} < 0$. This differential stabilization results in $W < E_{\mathrm{a}}$.

We explicitly illustrate the time-evolution of the energy of molecular system in Supplementary Figure 6e. During the tip approach, the system sequentially transitions from the initial state (structure 1) through intermediate structures 2 and 3, eventually reaching the transition state (structure 4). At this exact moment, the molecule instantaneously snaps to the final state (structure 7). Therefore, the reaction pathway effectively follows the sequence 1 → 2 → 3 → 4 → 7. Note that the remaining structures (5 and 6) pass by in an instant.

Finally, we discuss the relationship between our findings and established mechanochemical frameworks, such as the Bell model and Kramers' rate theory. In conventional mechanochemical models operating at finite temperatures, an applied mechanical force acts to lower the activation energy barrier, and the system subsequently overcomes the remaining, reduced barrier via thermal fluctuations ($k_{\mathrm{B}}T$). In sharp contrast, our experiments are conducted at a cryogenic temperature of 5 K, where thermal fluctuations are entirely negligible ($k_{\mathrm{B}}T \approx 0.4\,\mathrm{meV}$). Under these low-temperature conditions, the system possesses insufficient thermal energy to clear any remaining barrier. Therefore, to drive the structural transformation at 5 K, the effective activation barrier must be compressed completely to zero by the mechanical force of the approaching tip. The structural inversion thus takes place precisely at the mechanical instability point, where the energy barrier vanishes entirely (corresponding to the crossover point iii in Supplementary Figure 6d). This "vanishing barrier" mechanism does not contradict classical rate theories; rather, it delineates the absolute low-temperature limit of force-driven processes.

Supplementary Note 7. Additional observables during inversion

Supplementary Figure 8 presents additional observables recorded simultaneously during the force measurement shown in Fig. 3c (main text). Supplementary Figure 8a displays the frequency shift curve, which constitutes the raw data used to derive the force curve.

Supplementary Figure 8b displays the simultaneously recorded tunneling current. Although the sample bias was nominally set to 0 V, a slight instrumental offset resulted in a residual current. However, in all manipulation events, the current at the moment of inversion remained below 20 pA. Scanning Tunneling Spectroscopy (STS) on sumanene (Supplementary Figure 9) reveals peaks around -2.3 V and +2.5 V. The observed gap is consistent with the theoretical HOMO-LUMO gap of 4.6 eV calculated for a free sumanene molecule (*4*). Since the manipulation occurs within this large bandgap with negligible current, we rule out electron-induced mechanisms (e.g., resonant tunneling).

Supplementary Figure 8c shows the energy dissipation signal. No dissipation is observed during the elastic deformation in the approach path. A spike in dissipation appears only at the moment of the jump, which corresponds to the transient unlocking of the AFM Phase-Locked Loop due to the abrupt force change. Excluding this spike, the measured dissipation remains below 0.02 eV. This value is more than an order of magnitude smaller than W discussed in this paper, thereby validating the calculation of W from the conservative force curve hysteresis.

Supplementary Figure 8d shows the tip–sample potential energy curve derived from the force, where the energy difference between the approach and retraction curves at the closest approach corresponds to W.

Supplementary Note 8. Validity of W calculation from frequency shift

The inversion of sumanene is a single event occurring within a single oscillation cycle of the cantilever. However, experimentally measured quantities (frequency shift, dissipation) represent averages over many oscillation cycles. Given an integration time of 200 ms and an oscillation frequency of 18.5 kHz, each data point averages roughly 3700 cycles. Despite this averaging, the calculation of W from the hysteresis loop remains valid because the pre-jump (bowl-down) and post-jump (bowl-up) states are stable and maintained for long durations before and after the event. Consequently, the hysteresis loop formed by the averaged "forward" and "backward" frequency shift curves effectively reconstruct the hysteresis of the single-event force trajectory (as illustrated schematically in Fig. 1b).

It is worth noting that energy exchange can be discussed even if the tip is not oscillating (e.g., during an STM approach at V_s = 0 V). In such a case, the energy is supplied electrically by the piezo actuators. While it is theoretically possible to calculate energy from the voltage applied to the piezo, it requires precise calibration of the electromechanical coupling of the actuator, which is practically unfeasible compared to the direct force spectroscopy method using a cantilever.

One possible method to estimate the mechanical energy lost by a cantilever during a single oscillation cycle is to detect changes in amplitude. In this approach, oscillation of cantilever is driven by a constant excitation voltage with the amplitude feedback control disabled, and the amplitude change is monitored. The oscillating tip is then brought close to the sumanene to cause the inversion. The energy loss of the cantilever during the inversion is detected as a change in oscillation amplitude. Although the change in oscillation amplitude occurs instantaneously within a single cycle, the reduced amplitude persists for a duration proportional to the Q-factor of cantilever, providing a measurable time window. Unfortunately, current sensitivity is insufficient to detect such minute amplitude changes. The energy of the qPlus sensor oscillating at an amplitude of A = 100 pm is 62 eV. If 0.5 eV of energy is lost during inversion, the change in vibration amplitude would be 0.7 pm, which is below the current detection limit. In the future, repurposing high-speed amplitude-modulated AFM technology might enable the measurement of single-event energy cost.

Supplementary Note 9. Tip-induced molecular pickup

The tip-induced inversion is not the sole outcome of the manipulation experiments. In fact, molecular pickup by the tip also occurred. Supplementary Figure 10 shows a representative example of such a pickup event. Upon approaching a D molecule (red arrow in Supplementary Figure 10a), a jump is observed in the frequency shift (Supplementary Figure 10b). Subsequently, the retraction curve differs significantly from the approach curve. Subsequent STM imaging (Supplementary Figure 10c) confirms the disappearance of the molecule, leaving a vacancy. The short-range force curve (Supplementary Figure 10d) exhibits a jump followed by near-zero force, indicating the molecule was transferred from the surface to the tip. This suggests that a chemically active tip apex tends to pick up the molecule. Therefore, successful inversion requires a chemically inert tip (e.g., a molecule-terminated tip) that exerts repulsion without forming a strong bond.

Supplementary Note 10. Influence of tunneling effect in the molecular inversion

To rule out the possibility that the molecular inversion proceeds via nuclear tunneling—which would lower the apparent barrier—we evaluated the tunneling probability using the Wentzel–Kramers–Brillouin (WKB) approximation. According to the rate theory (*5*) and assuming a parabolic barrier with height V and width d, the critical barrier height V_C below which tunneling become dominant at temperature T is given by $V_C \approx 2m\pi^4(k_B T d)^2/h^2$. The inversion process requires the collective motion of carbon atoms (effective mass m^*) over a reaction coordinate distance (d). Our DFT calculation revealed that C atoms undergo a maximum displacement of 1.6 Å during inversion. Although the effective mass of cooperative motion of C atoms is expected to be significantly larger, we adopted the mass of single C atom (2×10^{-26} kg) as a lower bound. Substituting d = 1.6 Å, $m^* = 2 \times 10^{-26}$ kg, and temperature T = 5 K, V_C is estimated to be 6.7 meV. This value is two orders of magnitude smaller than the calculated activation energy of our system, E_a = 0.67 eV. Given that tunneling probability decays exponentially with $d\sqrt{Vm^*}$, we conclude that contribution of tunneling is negligible.

Supplementary Note 11. Contribution of electrostatic field

We evaluated the potential influence of the electric field on the reaction barrier. Our force spectroscopy measurements were performed at a sample bias of V_S = 0 V. Under this condition, the effective potential difference is determined by contact potential difference (CPD) between tip and sample. Measured CPD values were typically in the range of $|V_{CPD}| \approx 0.2 - 0.4$ V, depending on the tip condition. Assuming the maximum residual potential difference of 0.4 V and tip-molecule distance of 5 Å to reach transition state, resulting electric field is approximately E = 0.08 VÅ^{-1}. Sumanene possesses an intrinsic dipole moment μ of approximately 2.7 D due to its curved structure (*6*). In the planar transition state, this dipole moment vanishes, resulting in a net dipole change of $\Delta\mu \approx 2.7$ D. The maximum possible energy gain ΔU is given by $\Delta U = -E \cdot \Delta\mu \approx 0.045$ eV. This estimated energy gain is smaller than the energy difference ($E_a - W$) discussed in the main text. Therefore, the electrostatic contribution is also negligible.

Supplementary Note 12. Studies in other molecular systems

In our previous studies, we investigated repulsive-force-induced switching in other systems, such as the conformational change of a single NO molecule on Cu(110) (*7*) and the helicity switching of a graphene-nanoribbon (GNR)-fused helicene on Au(111) (*8*). For the GNR system, quantifying the intrinsic activation energy (E_a) via DFT is exceptionally challenging because the molecule is part of an extended ribbon, meaning that mechanical strain and stress are distributed widely throughout the structure. For the NO system, the measured input energy was roughly estimated to be 20–60 meV, which is lower than the calculated barrier of 125 meV, hinting at a potential sub-barrier process. However, because the NO molecule is light, the contribution of nuclear tunneling could not be rigorously ruled out. In contrast, the sumanene molecule studied here possesses an energy scale and molecular mass an order of magnitude larger than those of NO, making nuclear tunneling completely negligible (see Supplementary Note 10). Thus, the sumanene/Au(111) system provides an ideal and definitive platform to experimentally and theoretically validate the $W < E_a$ hypothesis. This energy-saving mechanism is a general feature of force-driven reactions where short-range repulsion and middle-range attraction coexist, rather than an effect unique to sumanene. It is expected that similar analyses will be conducted on other molecular systems in the future.

Supplementary Note 13. Trial of reverse molecular inversion

We systematically attempted to induce the reverse transition [from bowl-up (U) to bowl-down (D)] by approaching the tip to the center of a U-shaped sumanene molecule. However, no reverse inversion was observed. To trigger the reverse process, one would need to locally pull the center of the bowl upward, but the attractive force exerted by the tip was either insufficient in magnitude or lacked the necessary spatial confinement compared to the short-range repulsion. Notably, the absence of this reverse switching is a critical prerequisite for our experimental validation; during tip retraction after a successful D-to-U inversion, the tip inevitably traverses the attractive interaction regime. If the attractive force were strong enough to pull the molecule back into the D configuration, the net inversion could never be stabilized or observed. We also attempted to apply repulsive force to the outer rim of the U molecules to see if it could force a down-switching, but this resulted in complex, non-symmetric molecular rotations rather than a clean inversion. This highlights that applying a highly localized repulsive force exactly at the high-symmetry center of the molecule is essential to cleanly drive the structural transformation.

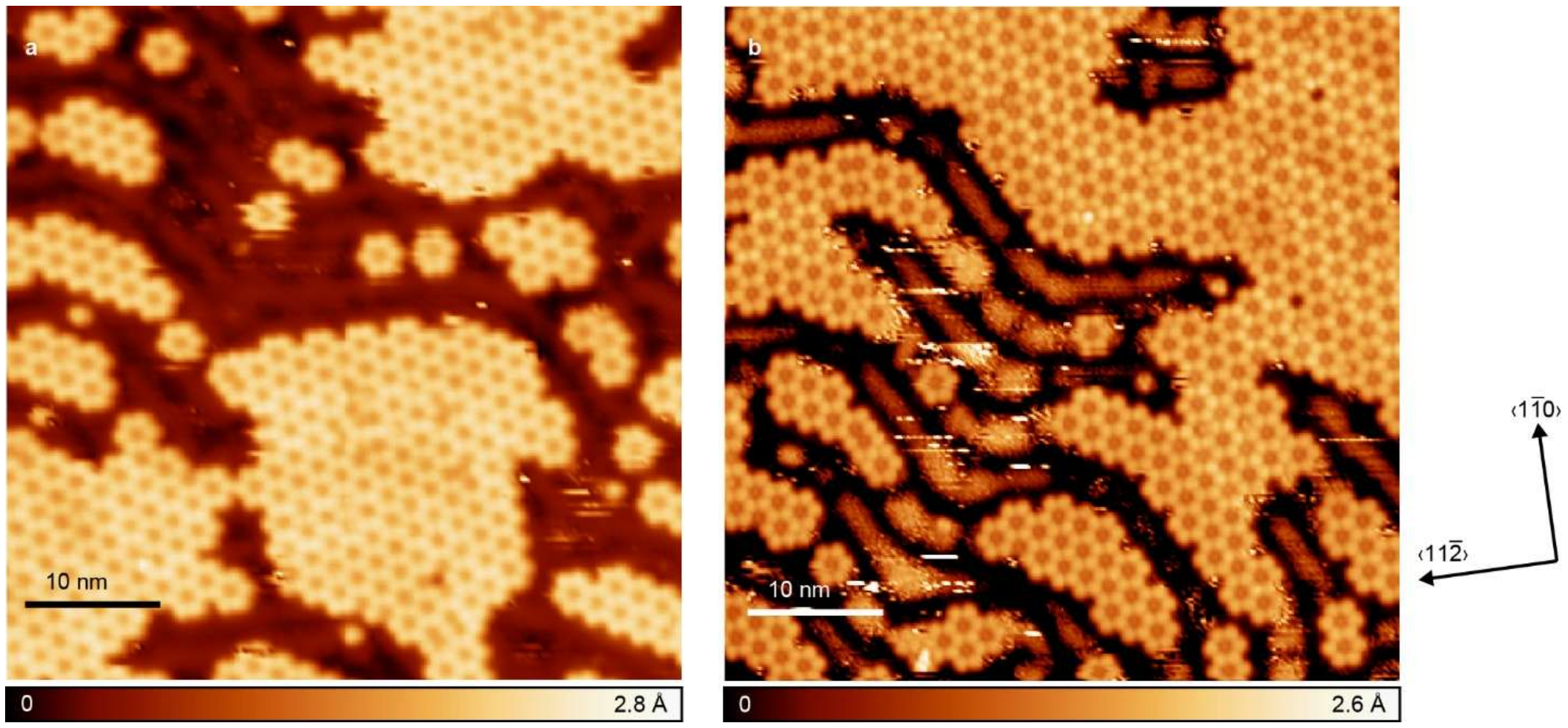

Supplementary Fig. 1. Large-scale STM images. STM images of sub-monolayer sumanene on Au(111) showing the herringbone reconstruction. Crystallographic directions of the Au substrate, estimated from the herringbone reconstruction, are indicated by black arrows. Acquired at (**a**) 5 K (V_S = -400 mV, I_t = 10 pA) and (**b**) 78 K (V_S = -500 mV, I_t = 10 pA).

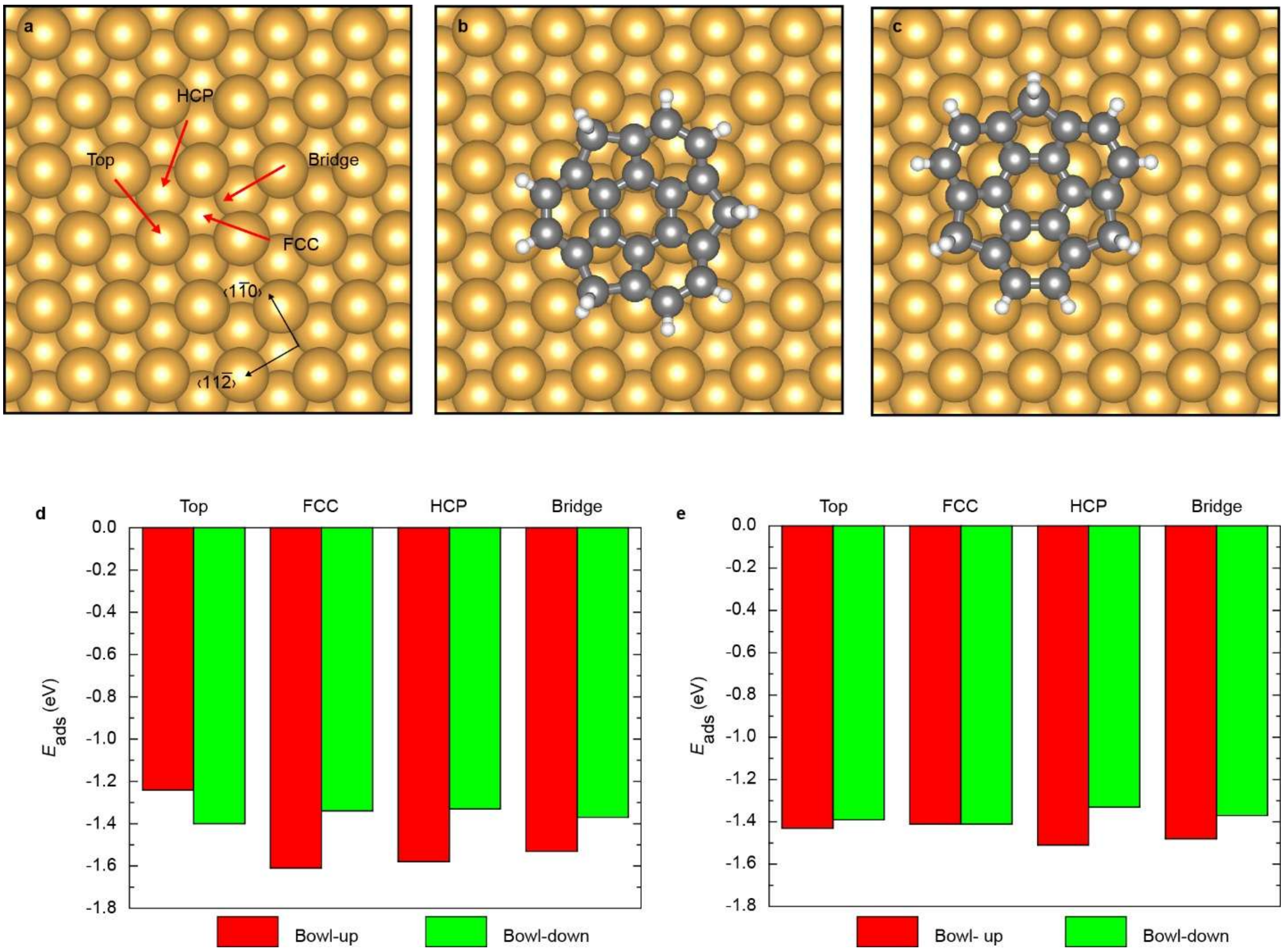

Supplementary Fig. 2. Adsorption states and energies of a single sumanene molecule. (**a**) High-symmetry adsorption sites on Au(111) considered in the calculations. (**b**) Adsorption geometry of a single bowl-up sumanene molecule in the 30° orientation at the FCC hollow site. (**c**) Adsorption geometry of a bowl-up one in the 0° orientation at the HCP hollow site. (**d, e**) Adsorption energies for the bowl-up and bowl-down single sumanene molecules at high symmetry sites in the (d) 30°and (e) 0° orientations. Source data are provided as a Source Data file.

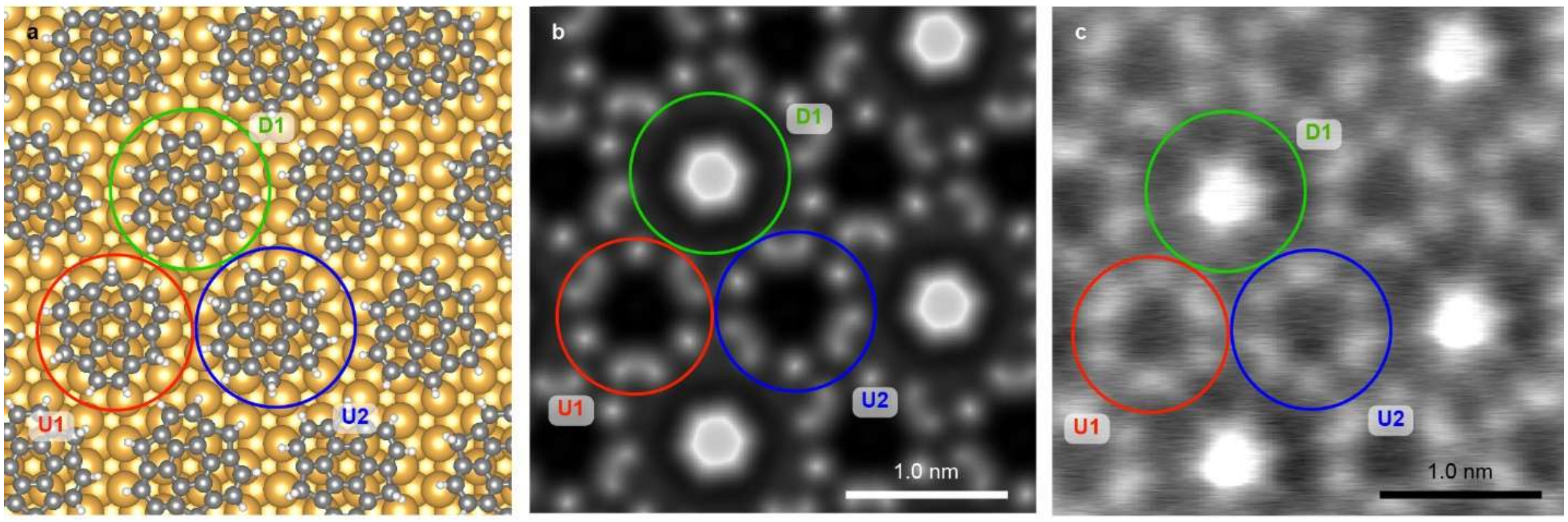

Supplementary Fig. 3. Structural optimization of the sumanene monolayer. (**a**) Calculated most stable structure for the 2U1D sumanene monolayer. (**b**) Simulated AFM image based on the structure shown in (a). (**c**) Experimental AFM image of the sumanene layer. Red, blue, and light green circles represent U1 (bowl-up, rotated by approximately 60°), U2 (bowl-up, not rotated), D1 (bowl-down, rotated by approximately 10°) sumanene molecules, respectively.

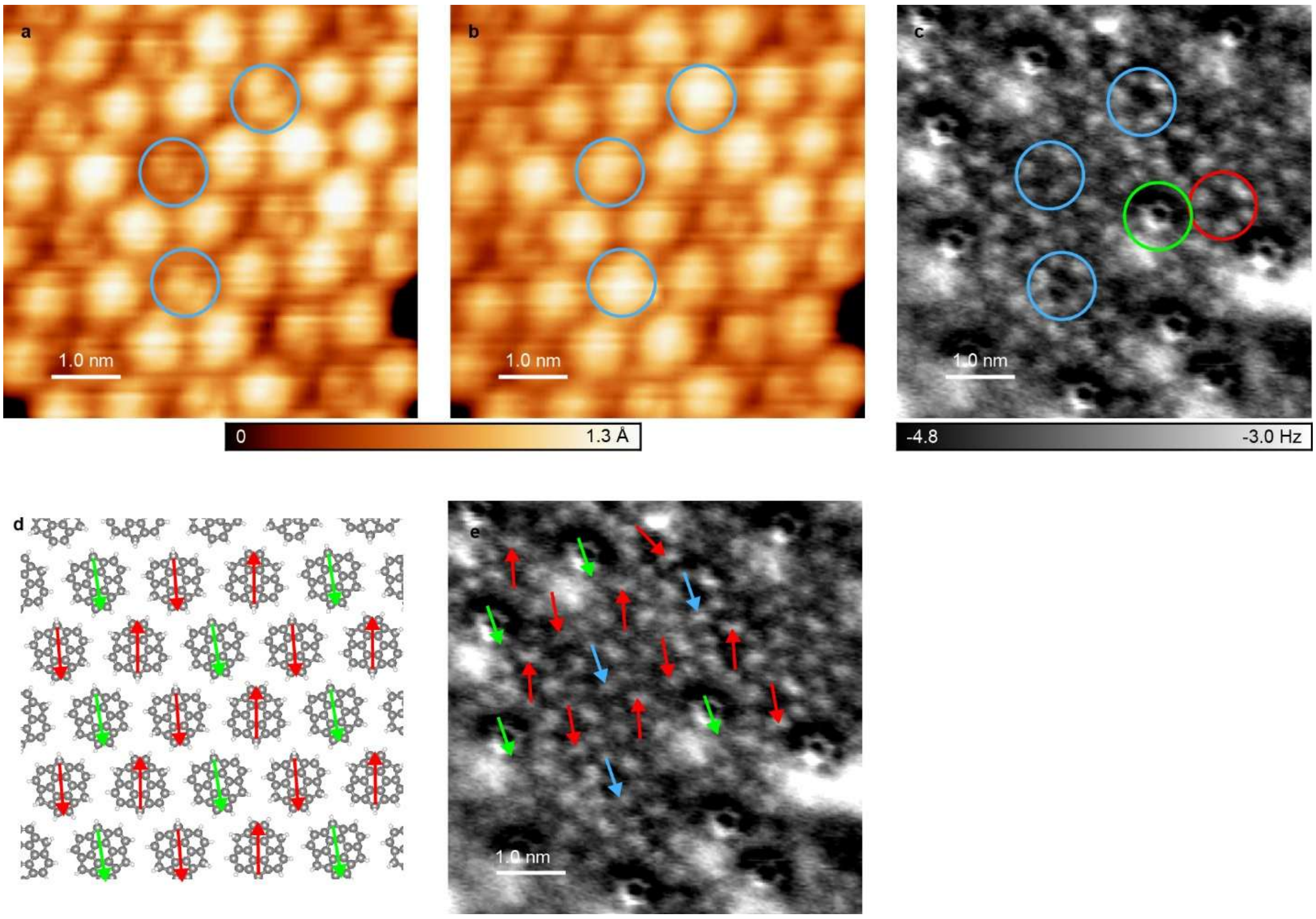

Supplementary Fig. 4. Structural identification of the inverted molecule. (**a, b**) STM images acquired before (a) and after (b) the manipulation (V_S = 100 mV, I_t = 20 pA). The light blue circles indicate the manipulated molecules. (**c**) AFM image of the same area acquired with a CO-terminated tip. Light blue, light green, and red circles indicate the manipulated, native bowl-down, and native bowl-up molecules, respectively. (**d**) Top-view model of the most stable structure. Arrows indicate the in-plane molecular orientation (defined from the 5-membered ring to the 6-membered ring). Red and light green denote bowl-up and bowl-down configurations, respectively. The Au substrate is omitted for clarity. (**e**) The AFM image from (c) overlaid with orientation arrows assigned based on the definition in (d). The light blue arrows indicate the orientation of the manipulated molecules.

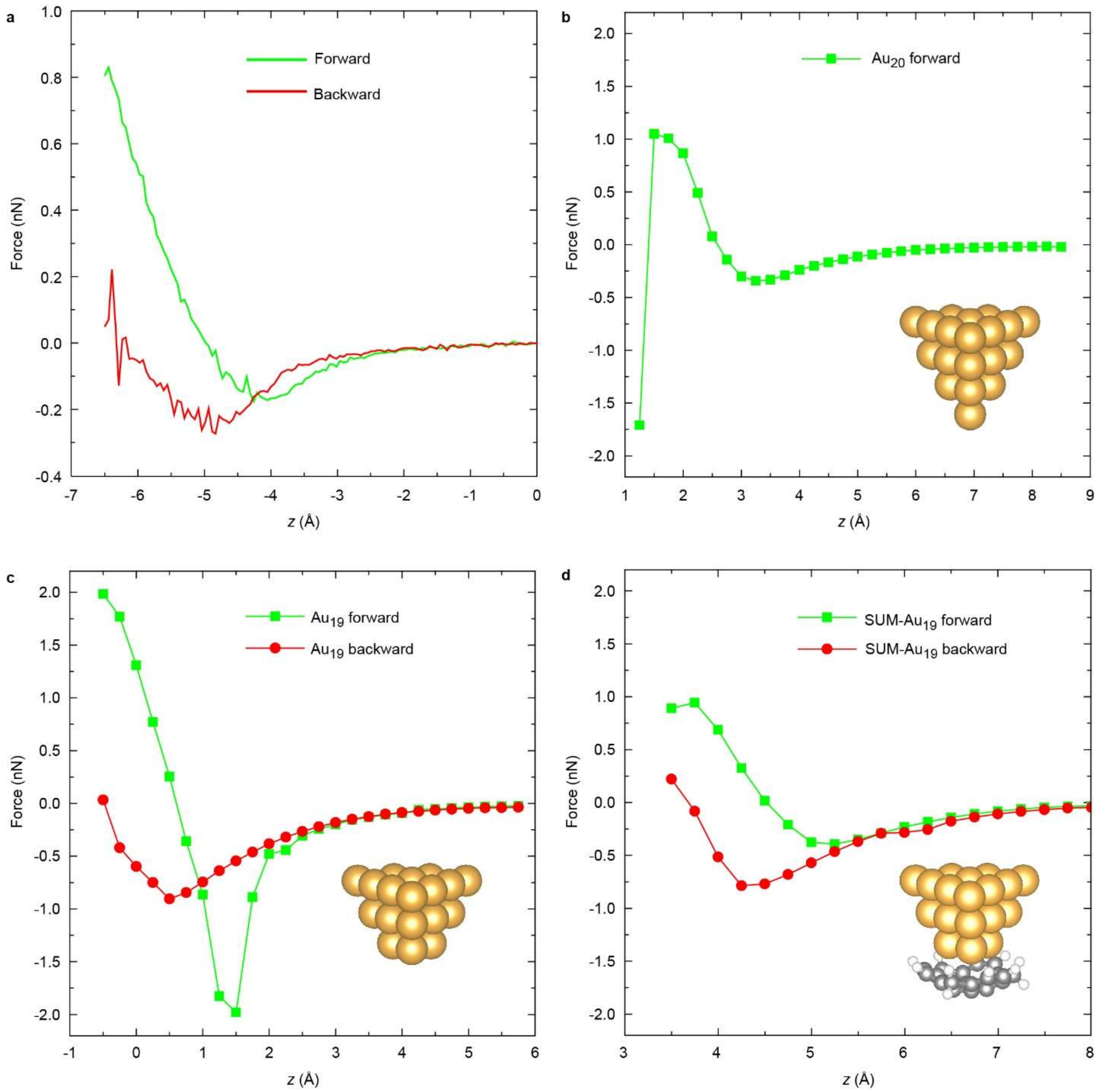

Supplementary Fig. 5. Comparison of experimental and theoretical force curves. (**a**) Experimental force curve obtained during inversion (same as Fig. 3c in the main text). Simulated force curves using (**b**) an Au_{20} tip, (**c**) an Au_{19} tip, and (**d**) a sumanene-terminated Au_{19} (SUM-Au_{19}) tip. Insets in (b), (c), and (d) show the respective tip models used for the calculations. Source data are provided as a Source Data file.

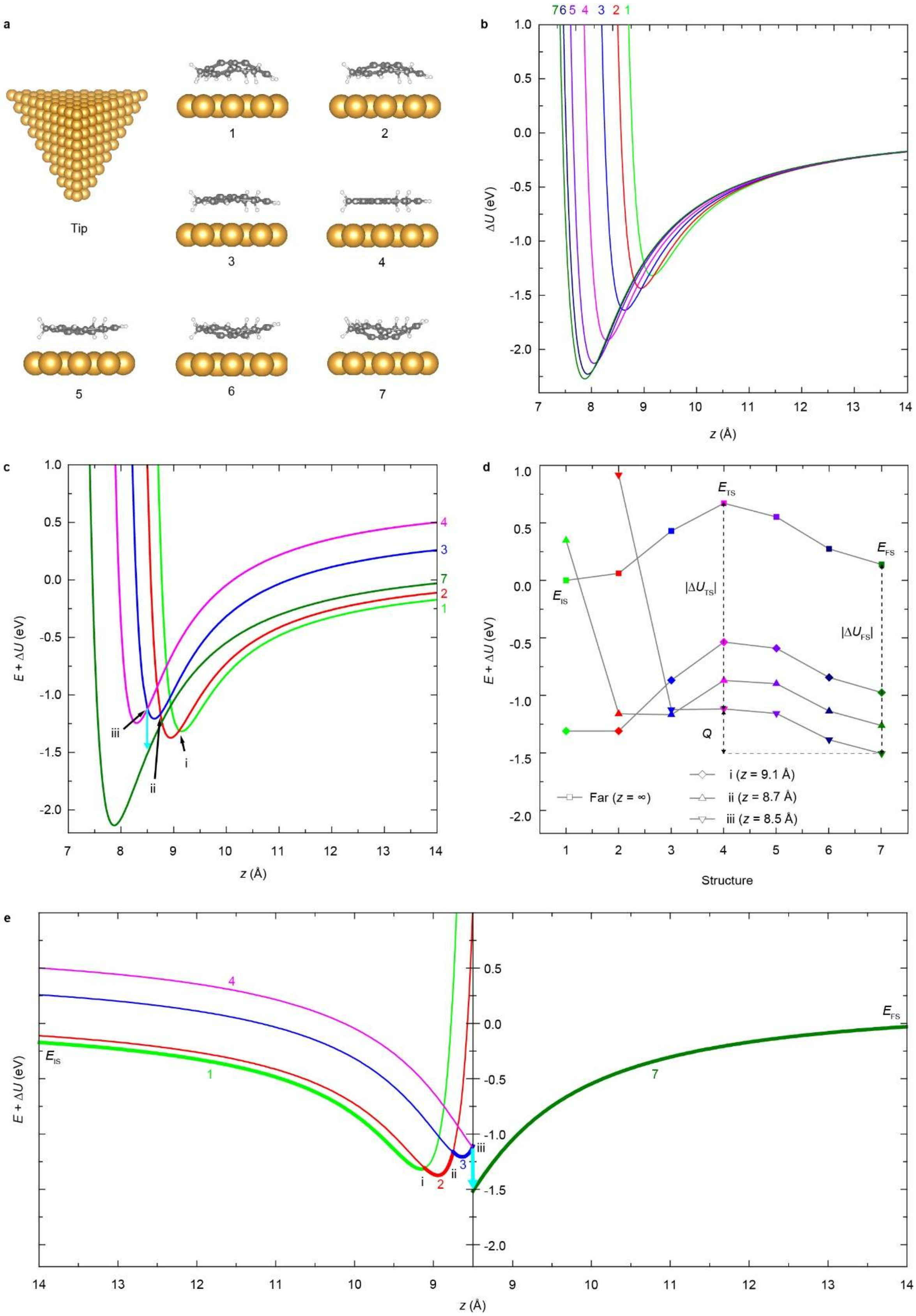
a
Tip
1
2
3
4
5
6
7
b
7 6 5 4 3 2 1
ΔU (eV)
z (Å)
c
E + ΔU (eV)
z (Å)
i
ii
iii
d
E + ΔU (eV)
Structure
E_TS
E_FS
E_IS
|ΔU_TS|
|ΔU_FS|
Q
Far (z = ∞)
i (z = 9.1 Å)
ii (z = 8.7 Å)
iii (z = 8.5 Å)
e
E + ΔU (eV)
z (Å)
E_IS
E_FS

Supplementary Fig. 6. Energetics of the inversion. (**a**) Geometries of the truncated tetrahedral "mesoscopic" Au tip model and the sumanene molecule in the molecular layer on Au(111) along the reaction coordinate for the inversion obtained using the NEB method. Structures 1, 4, and 7 represent the initial, transition, and final states, respectively, while structures 2, 3, 5, and 6 are intermediate states. Only the manipulated sumanene molecule and Au atoms in the first layer are shown. (**b**) Lennard-Jones potential (ΔU) between the molecular structures and the tip model shown in (a). (**c**) Total energy curves obtained by adding the Lennard-Jones potential shown in (b) to the intrinsic NEB energies. Intersections i, ii, and iii correspond to transitions between structures 1–2, 2–3, and 3–4, respectively. (**d**) Evolution of energy profile along the reaction coordinate. ΔU_{TS}, ΔU_{FS}, and Q are indicated by double-headed arrows. (**e**) The energy of the molecular system over time (following the sequential approach and retraction path from left to right). Source data are provided as a Source Data file.

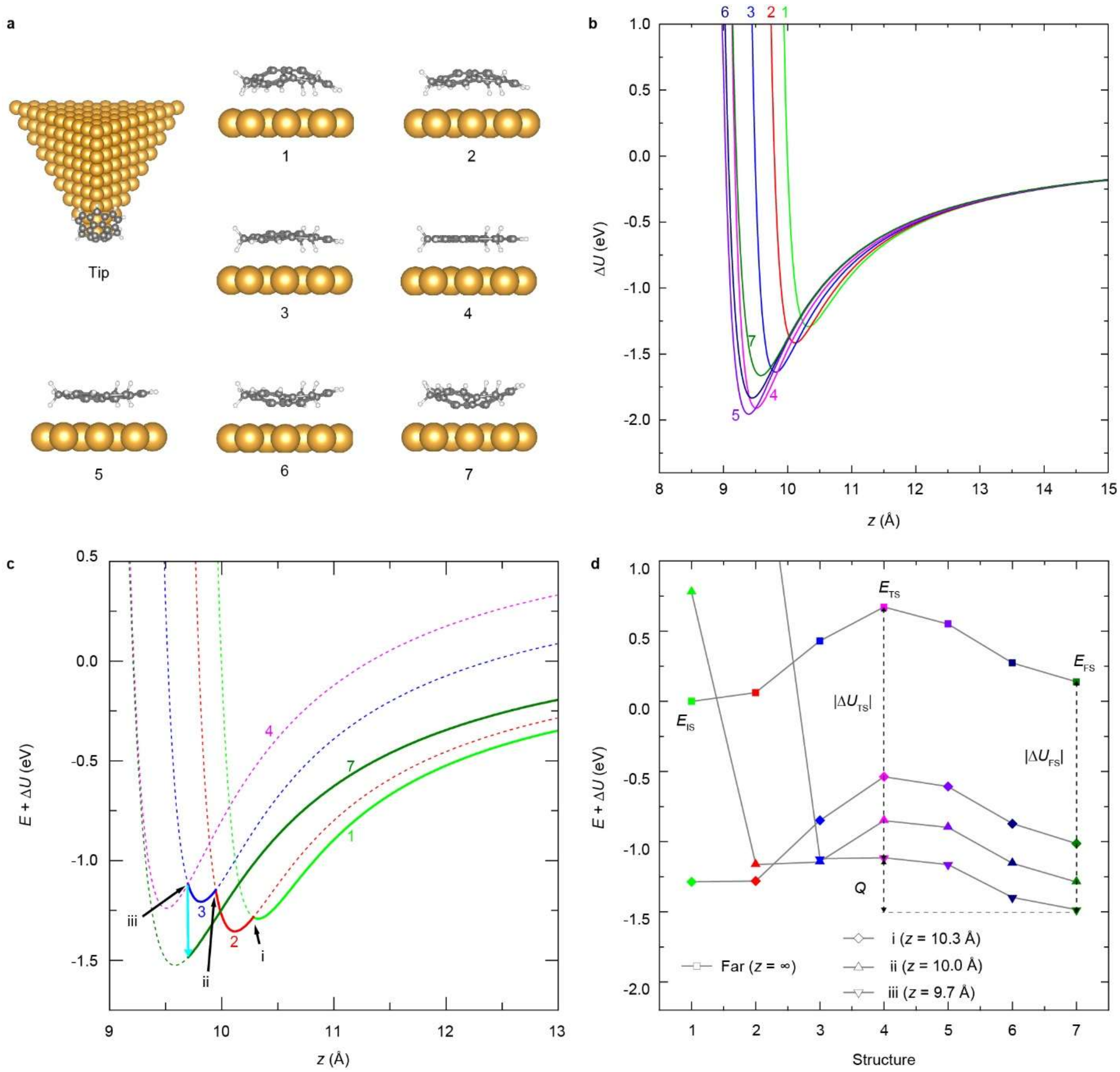

Supplementary Fig. 7. Energetics of the inversion. (**a**) Geometries of the sumanene-terminated Au tip (the truncated tetrahedral Au cluster composed of 219 atoms) model and the sumanene molecule in the molecular layer on Au(111) along the reaction coordinate for the inversion obtained using the NEB method. Structures 1, 4, and 7 represent the initial, transition, and final states, respectively, while structures 2, 3, 5, and 6 are intermediate states. Only the inverted sumanene molecule and Au atoms in the first layer are shown. (**b**) Lennard-Jones potential (ΔU) between the molecular structures and the tip model shown in (a). (**c**) Total energy curves obtained by adding the Lennard-Jones potential shown in (b) to the intrinsic NEB energies. Intersections i, ii, and iii correspond to transitions between structures 1–2, 2–3, and 3–4, respectively. (**d**) Evolution of energy profile along the reaction coordinate. ΔU_{TS}, ΔU_{FS}, and Q are indicated by double-headed arrows. This model yields $\Delta U_{\mathrm{TS}} < \Delta U_{\mathrm{FS}}$ ($\Delta U_{\mathrm{TS}} =$-1.79 eV vs. $\Delta U_{\mathrm{FS}} =$-1.62 eV), reproducing the $W < E_{\mathrm{a}}$ condition. Source data are provided as a Source Data file.

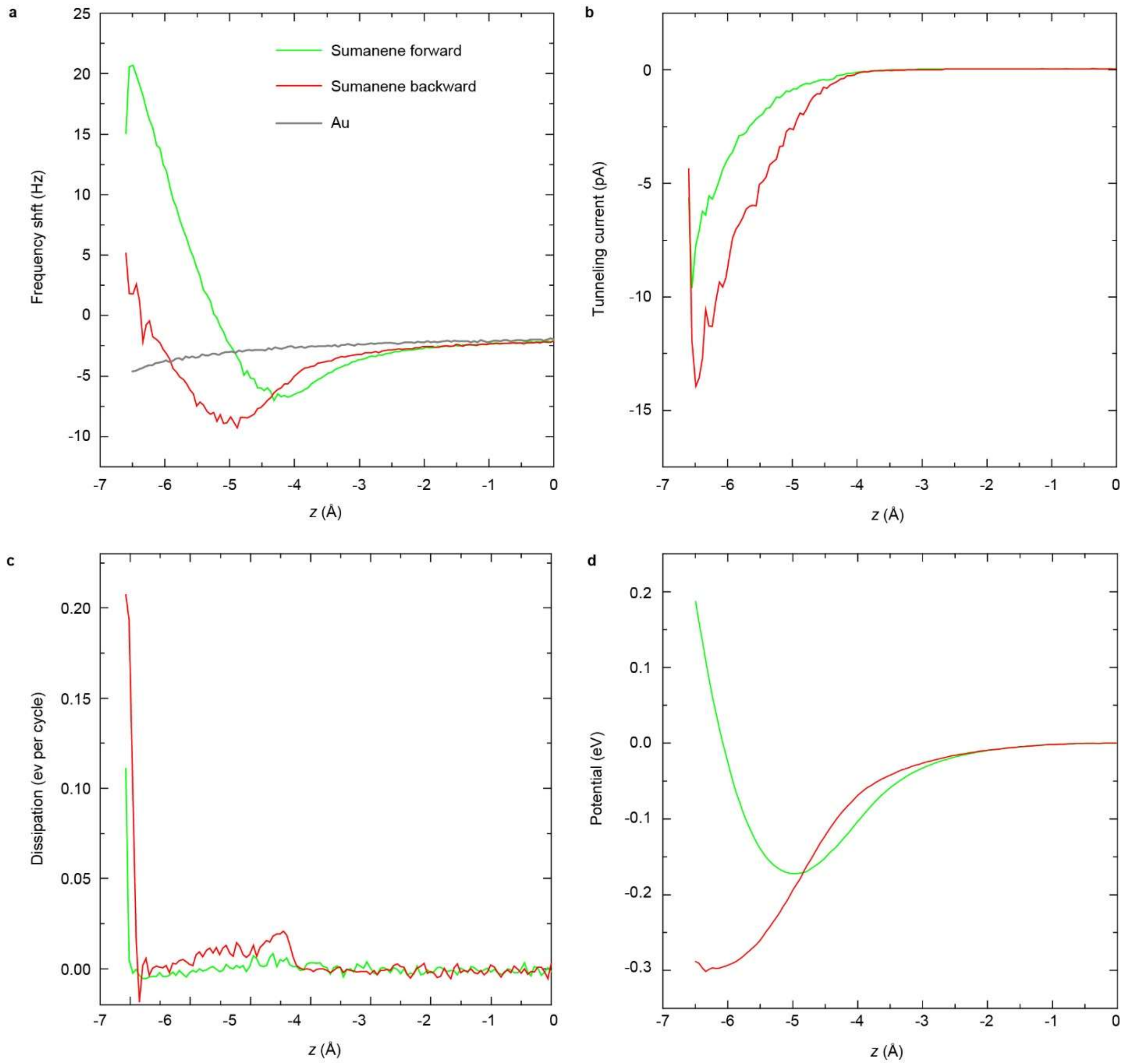

Supplementary Fig. 8. Additional observables recorded during the inversion. (**a**) Frequency shift curves corresponding to Fig. 3c in the main text. The gray curve indicates the background measured on the bare Au region. (**b, c**) Simultaneously recorded (b) tunneling current and (c) energy dissipation signals. (**d**) Tip–sample potential energy curve derived from (a). Source data are provided as a Source Data file.

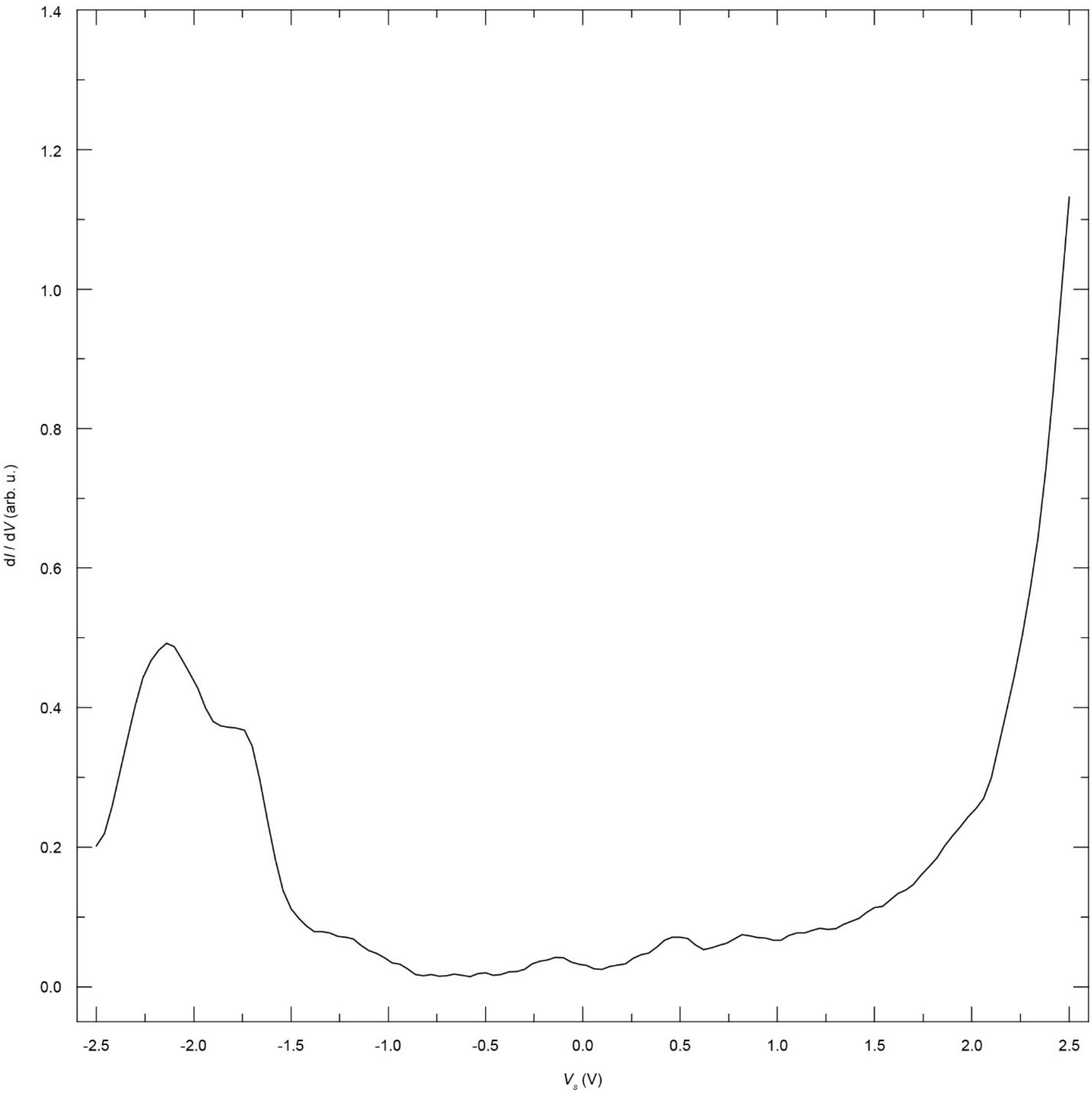

Supplementary Fig. 9. Electronic structure of sumanene. Scanning tunneling spectroscopy (STS) spectrum measured on a sumanene molecule. The spectrum was acquired at 85 K with lock-in detection (V_{mod} = 50 mV, f_{mod} = 97 Hz). Source data are provided as a Source Data file.

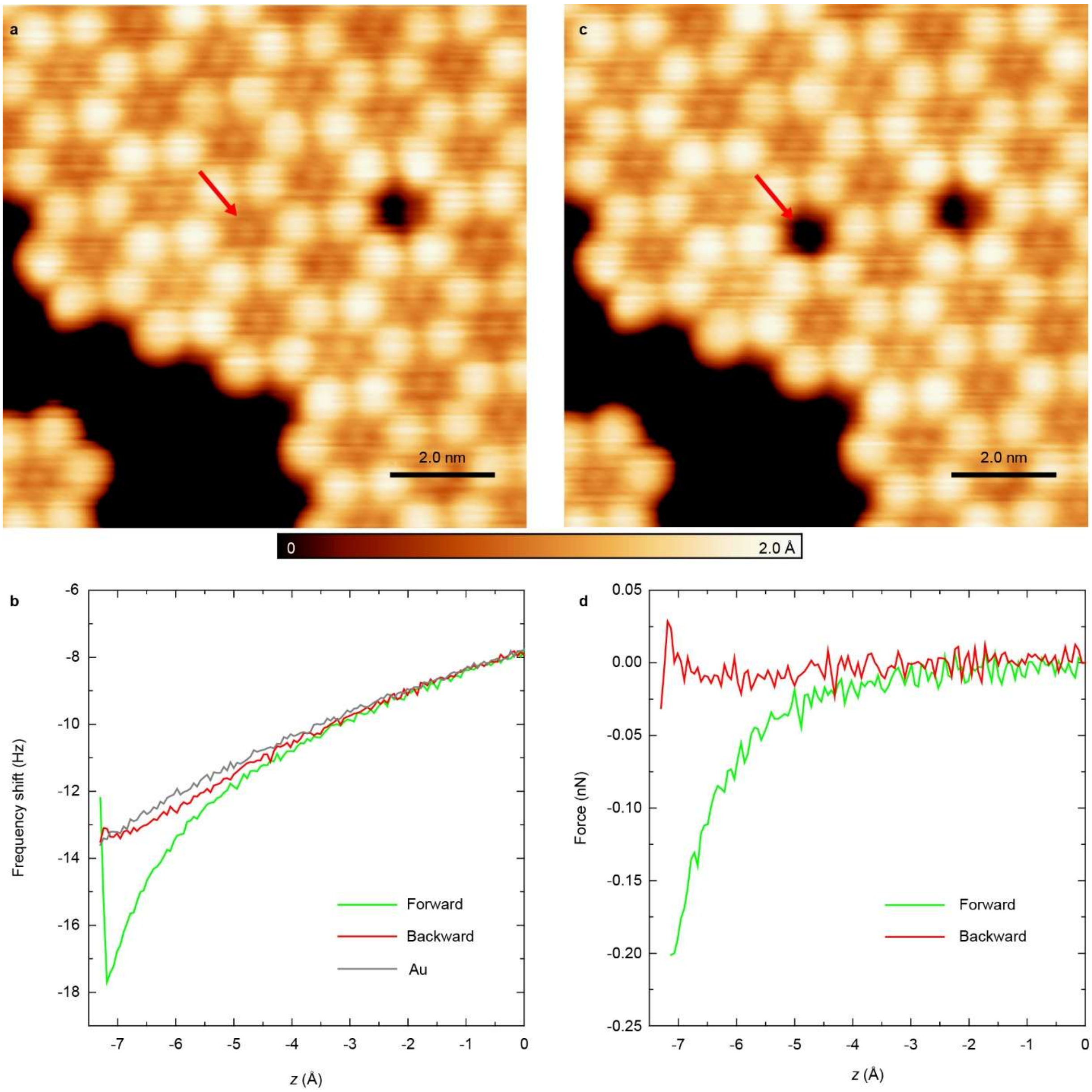

Supplementary Fig. 10. Example of unintended molecular pickup during a manipulation attempt. (**a**) STM image acquired before the manipulation (V_S = 100 mV, I_t = 30 pA). The red arrow indicates the target D molecule. (**b**) Frequency shift curve recorded during the approach to the target molecule. The gray curve indicates the background signal measured on the bare Au region. (**c**) STM image obtained after the measurement in (b) (V_S = 100 mV, I_t = 30 pA). (**d**) Short-range force curve derived from (b). Source data are provided as a Source Data file.

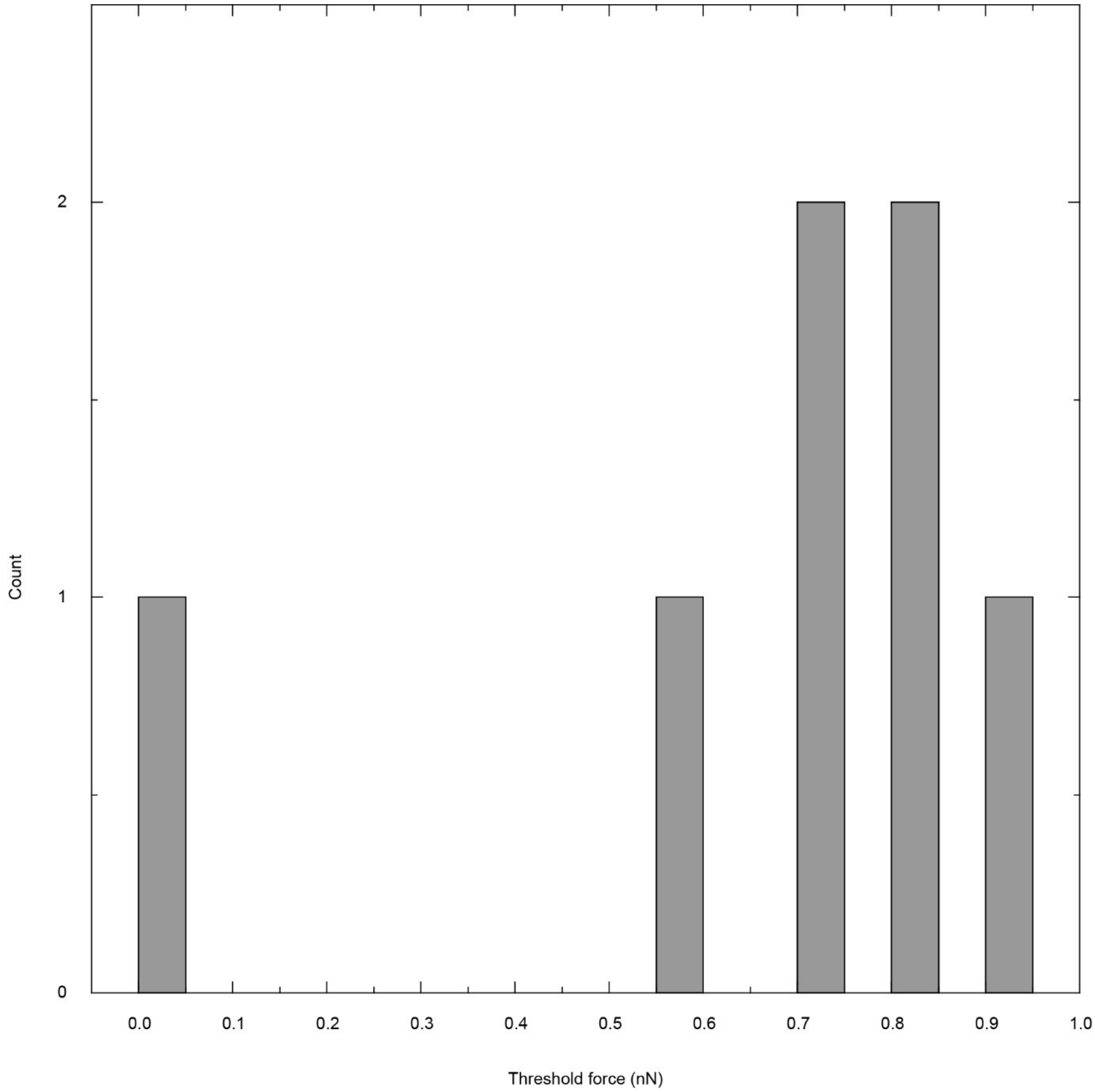

Supplementary Fig. 11. The threshold force at the moment of structural inversion. The values of the repulsive forces at the moment of jumps in the forward force curves are compiled into the histogram. Source data are provided as a Source Data file.

Supplementary References

1. Fujii, S., Ziatdinov, M., Higashibayashi, S., Sakurai, H. & Kiguchi, M. Bowl inversion and electronic switching of buckybowls on gold. *J. Am. Chem. Soc.* **138**, 12142–12149 (2016).
2. Jaafar, R. et al. Bowl inversion of surface-adsorbed sumanene. *J. Am. Chem. Soc.* **136**, 13666–13671 (2014).
3. Hapala, P. et al. Mechanism of high-resolution STM/AFM imaging with functionalized tips. *Phys. Rev. B* **90**, 085421 (2014).
4. Tan, Q. et al. Triazasumanene: an isoelectronic heteroanalogue of sumanene. *Bull. Chem. Soc. Jpn.* **91**, 531–537 (2018).
5. Hänggi, P., Talkner, P. & Borkovec, M. Reaction-rate theory: fifty years after Kramers. *Rev. Mod. Phys.* **62**, 251–341 (1990).
6. Shoji, Y. et al. Hexathioalkyl sumanenes: an electron-donating buckybowl as a building block for supramolecular materials. *Chem. Sci.* **8**, 8405–8410 (2017).
7. Shiotari, A., Odani, T. & Sugimoto, Y. Torque-induced change in configuration of a single NO molecule on Cu(110). *Phys. Rev. Lett.* **121**, 116101 (2018).
8. Ishii, A., Shiotari, A. & Sugimoto, Y. Mechanically induced single-molecule helicity switching of graphene-nanoribbon-fused helicene on Au(111). *Chem. Sci.* **12**, 13301–13306 (2021).
