# Supplementary material for: Force-driven single-molecule transformations through tip-mediated stabilization of transition states
Source: Nat Commun. 2026 Oct 23;17:10868. doi: 10.1038/s41467-026-78541-6 (PMC13694703; doi:10.1038/s41467-026-78541-6)
Supplement: Supplementary file 2 [file 41467_2026_78541_MOESM2_ESM.pdf]

Description of Additional Supplementary files

File Name: Supplementary Movie 1
Animation of the sumanene inversion process induced by a bare Au_{19} tip.

The trajectory was obtained from DFT calculations using an Au_{19} tip. The movie shows the sumanene molecule inverting its bowl structure due to the repulsive interaction with the approaching Au19 tip.

File Name: Supplementary Movie 2
Animation of the sumanene inversion process induced by a sumanene-terminated Au_{19} tip.

The trajectory was obtained from DFT calculations using a sumanene-terminated Au_{19} tip. The movie shows the surface sumanene molecule inverting its bowl structure due to the repulsive interaction with the approaching functionalized tip.
