# Supplementary material for: Force-driven single-molecule transformations through tip-mediated stabilization of transition states
Source: Nat Commun. 2026 Oct 23;17:10868. doi: 10.1038/s41467-026-78541-6 (PMC13694703; doi:10.1038/s41467-026-78541-6)
Supplement: Supplementary file 4 [file 41467_2026_78541_MOESM6_ESM.pdf]

nature portfolio

Peer Review File

Force-driven single-molecule transformations through tip-mediated stabilization of transition states

Corresponding Author: Dr Yoshiaki Sugimoto

This file contains all reviewer reports in order by version, followed by all author rebuttals in order by version.

Version 0:

Reviewer comments:

Reviewer #1

(Remarks to the Author)
The work by Iwata et al deals with performing a mechanical reaction on an organic molecule. Through means of AFM measurements and DFT calculations the authors demonstrate that you can mechanically invert a sumanene molecule upon the tip being in close distances to the molecule. There are indeed very few reports that demonstrate such type of reactions and even less that are scientifically credible, and the authors example is quite clever: a self-assembled sumanene layer seems to be the ideal system to probe such type of reaction. The experimental data and the simulations are of highest quality, the interpretations are reasonable and the manuscript reads smoothly. I support publication of this work in Nature Communications after the proper addressing of the following points:
1) Energetics balance (page 2 line 57): The authors state 'excluding tunneling-induced processes' – do the authors refer only to elastic tunneling processes? I do not think the continuation sentence is fully correct 'and the injected energy is completely dissipated after the reaction'. One counter example is when new bonds are created. I suggest improving these sentences.
2) In the theoretical framework, the authors assume (without any explanation) that TS is reached at the closest tip-sample distance. This is not evident nor necessary, I would argue. Please add a sentence in the manuscript explaining this logic.
3) In the DFT calculations, the authors utilize a sumanene-terminated tip, whereas the experimental results are for a metallic tip (not state in the caption nor main text). Why do they have this difference in tip termination for the comparison between theory and experiment?
4) What is the estimated error in the activation energy using the NEB method? I think it is important to include this for the readership.
5) In the SI the authors state 'Force spectroscopy measurements were also acquired at zero bias (Vs = 0). But this does not guarantee that you are at the minimized electrical field. Do the authors have examples of datasets where the bias is altered by + or – 10 mV? Do they observe the same mechanical inversion? The authors should also state what the LCPD of their tip was. That would allow readership to judge their measurements in full context.
Minor
- Page 2 line 68: Please add a verb after E_FS … the energies. I would use 'be'.

Reviewer #2

(Remarks to the Author)
The manuscript from Sugimoto and coworkers reports the inversion of a sumanene molecule on gold surface using qplus AFM and compares the mechanical work performed by the tip and the activation energy of the reaction. Although the experimental results are well performed, the explanation of the manipulation is not convincing, and maybe quite misleading. There has been quite a lot of representative papers in this area and the authors are recommended to cite those papers and discuss how the mechanism is different. After careful evaluation, I do not believe that the present manuscript meets the standard required for publication in Nature Communications, and I therefore recommend a major revision.
My main concern is about how the authors connect the experimental results with W, which the authors define as the work done by the tip on the molecular system. First, the authors claims that W corresponds to the mechanical energy lost by the oscillator, but only Figure S7(c) shows the dissipation and the discussion on it is not enough. On the contrary, the authors attribute W to the hysteresis of the forward and backward. This is quite confusing. The force spectrum measures the force between tip and sample and the displacement is for tip, thus the integration is the work on tip. The hysteresis represents the

different interaction on tip by the two different molecules (up/down) and this cannot be linked to the work on molecule. For another example, considering the force spectrum measured on two kinds of SnPc molecule on surface with Sn pointing up and down at the same z range, the difference between the two force spectra cannot be relative to the energy pushing a Sn atom through the molecule. I recommend the authors to reconsider the theoretical model to explain the experimental finding based on the work from the guys like Ernst Meyer, Peter Grütter, or Alexander Schwarz. In my opinion, the authors can focus on the forward force spectrum alone and remove the background force (not on gold, but the hole in the assembling structure) to obtain the critical energy where the inversion of molecule happens, then they may get more convincing results.
Besides the above issue, a more detailed discussion on Fig. 4 and Fig. S7 is also needed. If I understand it correctly, the Figure 4d shows the energy at different height on different structures, so the intersections should refer to tip height to make it easier for readers to understand. The intersections are points in Fig. 4c so it is hard to understand it easily. Another question is that there should be 6 intersections between 7 structures, so the authors can show the rest to give the readers a full picture.

Reviewer #3

(Remarks to the Author)
In this article, the authors revealed that the mechanical work W (measured by AFM) is smaller than the activation energy Ea (obtained by NEB calculations), implying energy savings in the configuration switching of a bowl-shaped molecule. The authors have previously reported a similar work, i.e., repulsive-force-induced configuration switching of a single NO molecule on Cu(110) in Phys. Rev. Lett. 121, 116101 (2018). The idea is valuable in the context of mechanochemistry. However, several issues need to be carefully addressed, and I think the current version is below the requirements of Nat. Commun.

1. The observed difference between W (~0.50 eV) and Ea (~0.67 eV) is about 0.17 eV. The authors should discuss whether this difference lies within the combined experimental and computational error margins. Specifically:
For W extraction from force curves: the choice of zero point, the correction for non-conservative forces, and the integration limits all introduce uncertainties.
For Ea from DFT: The authors should know the DFT can only treat ideal situations, at zero temperature and pressure. As W and Ea are respectively from experimental and computational approaches, is it scientifically correct to deduce the value of 0.17 eV as the energy savings? This is central, and clarification on this point is critical for the main findings of this work.

2. In the authors' previous work (Phys. Rev. Lett. 2018, 121, 116101) and works from other groups, were a similar comparison between W and Ea performed? If so, was the W < Ea behavior already observed, or is it unique to the present molecular system (sumanene on Au(111))? Please clarify whether the energy-saving phenomenon is a general feature of repulsive-force-induced switching or specific to the bowl-shaped molecule.

3. The authors claimed that the contributions of tunneling and electrostatic are negligible. How do the authors assess the contribution of inelastic energy dissipation during the jump? This is particularly relevant because the hysteresis loop area includes both conservative and dissipative components.

4. The authors claimed that mechanical interactions allow a portion of the input energy to be recovered as work during the tip's motion. However, the tip is an oscillator; its mechanical energy change is governed by the driving force and dissipation. The potential energy landscape of the tip–molecule system may change before and after the inversion, resulting in different force–distance curves on approach and retraction. Consequently, the net mechanical energy loss of the cantilever is reduced.

5. The discussion does not relate the central finding W < Ea to any established theories of reactions (e.g., Bell model, Kramers' rate theory). Even though reference [47] cites the Kramers review, it is not discussed in the text. How does the present result fit into or differ from the classical framework where an applied force lowers the effective barrier but still requires an energy input at least equal to the remaining barrier height?

6. In the beginning of the results section, the STM image (Fig. 2B) labels two distinct features as U and D, but to a reader these merely appear as different bright spots. The authors should first schematically explain the two molecular orientations (bowl-up vs. bowl-down) in Fig. 2A, and then use high-resolution AFM images and DFT calculations to unambiguously assign the structures.

7. The manipulation experiments were performed exclusively on the D (bowl-down) molecules. After converting D to U, can the reversed process be achieved (e.g., by pulling the tip or using a different approach)? Can the U (bowl-up) molecules also be inverted to the D configuration by applying repulsive force? Demonstrating reversibility would greatly strengthen the claim of controlled mechanical switching. Please discuss whether such experiments were attempted and, if not, state the limitations.

8. The main text lists references up to [36]. References [37]–[48] should appear in the supplementary materials but are not clearly marked. Please indicate which references belong to the main text and which to the SI, or renumber them accordingly to avoid confusion.

Version 1:

Reviewer comments:

Reviewer #1

(Remarks to the Author)
In their rebuttal, Iwata et al. properly addressed my concerns and seem to clarify the concerns of the other reviewers. I agree with the authors that showing that a mechanochemical reaction can happen despite having a larger activation energy barrier is quite interesting. To me, the manuscript is ready to be published.

Reviewer #2

(Remarks to the Author)
I have read the reply to me, and I think that the revised manuscript only solves minor issues. Although the experiments were well-performed and the presentation has been improved, the explanation on the theoretical model is still not convincing. Therefore, it is still below the requirements of Nat. Commun. in this version.
First, there are still some minor issues. The tip used in all images should be denoted to show which kind of tip is used. I didn't find any experimental results which confirm the reversion by a metal tip, while there are only theoretical results in the SI. If the molecules cannot be inversed by metal tip experimentally, what was the analyzation for? In addition, the authors claim that the tip is sumanene-terminated, why would an Au tip be used in Fig. S6 for energy analyzation?
The schematic in Fig. 1A is not suitable because the three tip positions are quite misleading that they look like in the same oscillation period. If I understand it correctly, they are not in the same oscillation period and they only represent "far-medium-close". Otherwise, the discussion on dissipation should be provided. The schematic may need to be improved. By the way, the Fig 2A looks like the molecule can be reversed by a metal tip, which may also be modified or the author should show the relative experimental results at least.
The above issues are solvable. The main issue is the theoretical model and in the following I have to explain my main concern.
I still have questions on the comparison between W and Ea, which is the central find of this manuscript. I also agree that the potential energy landscape of the tip–molecule system changes before and after the inversion. The comparison between the forward and backward energy spectra is not related to the energy barrier of the inversion.
The authors claim that at the lowest tip position the inversion emerges. Therefore, the interaction force and energy between tip and molecule at that position is quite important to evaluate the barrier of the inversion. I understand that this point of view is "conventional" for the authors but it has been correct in most cases and well-accepted in the community. The effort on removing the background force is a known choice to get quantitative analyzation on the barrier, which is what I recommended in the first time.
The last issue is about the energy conservation. There are two kinds of energy conservation at least. The first is the oscillation of the tip. If there is any dissipative interaction between tip and sample, it requires more energy to keep the oscillation of the tip. This has been proven by the authors that the dissipative interaction is negligible. In this case, the second part is more important. When the tip approaches to sample, the potential energy between tip and sample is changing but the kinetic energy of the tip or sample is unchanged. Where does the energy go or come from? It is the tip holder (if it is the tip which moves, otherwise this will be the sample holder). The interaction between tip and sample does the positive energy and the tip holder does the negative energy, or vice versa. Thus, the difference between the two spectra (W) has nothing to do with the energy transfer to the molecule. The authors should not use the conservation of energy in the response to my concern about the work on molecule.

Reviewer #3

(Remarks to the Author)
In the revised version of manuscript, I see that the authors have addressed all the concerns and provided convincing responses. I therefore recommend acceptance of it for publication in Nature Communications.

Version 2:

Reviewer comments:

Reviewer #2

(Remarks to the Author)
The current version could be published without any revision.

Open Access This Peer Review File is licensed under a Creative Commons Attribution 4.0 International License, which permits use, sharing, adaptation, distribution and reproduction in any medium or format, as long as you give appropriate credit to the original author(s) and the source, provide a link to the Creative Commons license, and indicate if changes were made.
In cases where reviewers are anonymous, credit should be given to 'Anonymous Referee' and the source.
The images or other third party material in this Peer Review File are included in the article's Creative Commons license, unless indicated otherwise in a credit line to the material. If material is not included in the article's Creative Commons license and your intended use is not permitted by statutory regulation or exceeds the permitted use, you will need to obtain permission directly from the copyright holder.
To view a copy of this license, visit https://creativecommons.org/licenses/by/4.0/

Response to the Editor and Reviewers

Thank you very much for reviewing our manuscript entitled "Single-molecule structural transformation by mechanical force" and for providing us with the valuable opportunity to submit a revised version. We also sincerely thank the three reviewers for their time, constructive feedback, and deep insights.

We have carefully addressed all the concerns raised by the reviewers. In particular, we have substantially refined our theoretical framework to clarify the definition of W, corrected a conceptual description regarding energy input, and enhanced the Supplementary Information (SI) with multiple new sections and figures (including a discussion on other molecular systems, a summary of force thresholds, and a temporal evolution of energy in molecular system). We believe these revisions have eliminated any previous misunderstandings and have conclusively strengthened the core hypothesis ($W < E_a$) of our work. We hope that the revised manuscript is now fully suitable for publication in *Nature Communications*.

Reviewer #1 (Remarks to the Author):

The work by Iwata et al deals with performing a mechanical reaction on an organic molecule. Through means of AFM measurements and DFT calculations the authors demonstrate that you can mechanically invert a sumanene molecule upon the tip being in close distances to the molecule. There are indeed very few reports that demonstrate such type of reactions and even less that are scientifically credible, and the authors example is quite clever: a self-assembled sumanene layer seems to be the ideal system to probe such type of reaction. The experimental data and the simulations are of highest quality, the interpretations are reasonable and the manuscript reads smoothly. I support publication of this work in Nature Communications after the proper addressing of the following points:

We sincerely thank Reviewer #1 for the highly positive evaluation of our work. We are grateful for the reviewer's recognition that our self-assembled sumanene layer is an ideal system to probe single-molecule mechanochemistry, and that our experimental data and simulations are of the highest quality, reasonable, and smoothly written. Below, we provide our point-by-point responses and details of the corresponding revisions.

1) Energetics balance (page 2 line 57): The authors state 'excluding tunneling-induced processes' – do the authors refer only to elastic tunneling processes? I do not think the continuation sentence is fully

correct ‘and the injected energy is completely dissipated after the reaction’. One counter example is when new bonds are created. I suggest improving these sentences.

We appreciate the reviewer's insightful comment. The phrase "tunneling-induced processes" was originally intended to mean the *nuclear* tunneling effect of the molecule itself during the reaction pathway (where a reaction might proceed via tunneling even if the external energy input is below the classical activation barrier). To avoid any ambiguity or misunderstanding, we have removed the phrase "*(excluding tunneling-induced processes)*" from the main text.
Furthermore, to clearly illustrate the fundamental difference between conventional excitation methods and our mechanical approach, we have rewritten this section and also updated the Abstract as follows:

Revised text in Main Text (Page 2, Line 56):
In conventional single-molecule reactions triggered by inelastic electron tunneling or photon absorption, the external energy is supplied in a one-way process. After the reactions, the input energy cannot be recovered, with the excess energy being dissipated into the substrate as heat. Consequently, the total energy consumption in these excitation processes inevitably exceeds the activation energy (E_a).

Revised text in Abstract:
We discover that the mechanical energy required to trigger this conformational change is significantly lower than the theoretical activation energy, in contrast to conventional activation methods such as tunneling electrons and photons. This energy-saving reaction proceeds because van der Waals attraction from the mesoscopic tip body stabilizes the transition state more effectively than the final state.

2) In the theoretical framework, the authors assume (without any explanation) that TS is reached at the closest tip-sample distance. This is not evident nor necessary, I would argue. Please add a sentence in the manuscript explaining this logic.

We agree with the reviewer that this deterministic assumption requires explicit justification. Experimentally, the sample surface is gradually brought closer to the vertically oscillating tip. When the distance between the sample and the tip (specifically at the lowest turnaround point of the oscillation) is large, no reaction occurs. When the structural inversion is eventually captured at a certain critical Z-distance, the reaction must have been triggered at the exact moment when the tip reached its closest approach to the sample during that specific oscillation cycle. Because our experiments are conducted at a cryogenic temperature of 5 K, thermal fluctuations are negligible, and

the reaction occurs deterministically with respect to the tip position. This precise moment of instantaneous reaction is what we sketch as the transition state (TS) configuration in Figure 1A. Following the reviewer's suggestion, we have added this explanatory logic to the main text:

Added text in Main Text (Page 2, following Line 74):
We assume that the sample is gradually brought closer to the oscillating tip. If the distance between the sample and the tip at the lowest turnaround point of the oscillation is too large, the reaction does not occur. When the reaction is observed at a specific distance, it must occur at the exact moment when the tip reaches its closest approach to the sample during the oscillation. The reaction proceeds deterministically with respect to the tip position at a sufficiently low temperature. This instantaneous moment of reaction corresponds to TS configuration sketched in Fig. 1A.

Note that the possibility of the reaction proceeding via an altered reaction coordinate (different from the unperturbed, tip-free TS geometry) is already thoroughly discussed in Section 1 of the Supplementary Information ("General condition for $W < E_a$ in tip-induced reactions").

3) In the DFT calculations, the authors utilize a sumanene-terminated tip, whereas the experimental results are for a metallic tip (not state in the caption nor main text). Why do they have this difference in tip termination for the comparison between theory and experiment?

We would like to clarify that in our DFT calculations, we systematically investigated both bare metallic tips (Au_{20} and Au_{19}) and a sumanene-terminated tip, all of which successfully reproduced the molecular inversion within the repulsive force regime as detailed in Section 5 of the Supplementary Information ("DFT simulation of tip-induced inversion ") and Fig. S5.
We chose to present the sumanene-terminated tip in Figure 3 of the main text because its calculated force curve yielded the closest quantitative agreement with our experimental results. Crucially, the intramolecular contrast patterns observed in our experimental STM images (Figs. 3A and 3C) suggest that the tip apex was already functionalized by a molecule, most likely due to the inadvertent pickup of a sumanene molecule during prior manipulation attempts.
As discussed in Section 9 of the Supplementary Information ("Tip-induced molecular pickup") and Fig. S9, clean metallic tips exhibit a strong tendency to pick up sumanene molecules from the gold surface. In the field of NC-AFM, it is well established that modifying the tip apex to make it chemically inert (similar to CO-functionalization) is essential to suppress such destructive pickup events, thereby allowing the tip to stably enter the repulsive regime and manipulate molecules non-destructively.
To explicitly clarify this tip condition for the readers, we have added the following discussion to the main text:

Added text in Main Text (Page 4, Line 144):

The intramolecular contrast in the STM images (Figs. 3A and 3C) suggests the use of a molecule-functionalized tip, as we suspect a sumanene molecule was inadvertently picked up by the tip during the experiments. As discussed in Section 9 of the Supplementary Information and Fig. S9, a bare metallic tip tends to pick up sumanene molecules from the surface.

4) What is the estimated error in the activation energy using the NEB method? I think it is important to include this for the readership.

We agree that discussing the computational error margin is highly important for the readership. Quantifying the precise error in the calculated activation energy is complex because it is influenced by multiple factors, such as the choice of the exchange-correlation functional. Additionally, due to the exceptionally large size of the supercell required for this surface system, it is computationally prohibitive to systematically vary parameters like the number of intermediate images.

To address this, we systematically performed NEB calculations using three different supercells—(6 × 6), (6 × 6√3), and (12 × 12)—yielding activation energies of 0.744, 0.694, and 0.671 eV, respectively. By extrapolating these values, we estimate the activation energy in the isolated limit to be approximately 0.61 eV. This extrapolation effectively quantifies the error margin associated with periodic boundary conditions.

We have incorporated this explanation into the supplementary information on Page 2:

Added text in Supplementary Information (Materials and Methods):

We obtained the activation energies of 0.744, 0.694, and 0.671 eV for the three supercells, respectively. By extrapolation, we estimate a value of 0.61 eV for the isolated limit. This estimated quantifies the error associated with the use of the periodic boundary conditions.

5) In the SI the authors state 'Force spectroscopy measurements were also acquired at zero bias (Vs = 0). But this does not guarantee that you are at the minimized electrical field. Do the authors have examples of datasets where the bias is altered by + or – 10 mV? Do they observe the same mechanical inversion? The authors should also state what the LCPD of their tip was. That would allow readership to judge their measurements in full context.

We would like to clarify that our single-molecule inversion experiments were conducted exclusively at a sample bias of $V_s = 0$ V. Because this manipulation requires bringing the tip extremely close to the molecule into the deep repulsive regime, applying even a tiny voltage like ± 10 mV would induce

an enormous tunneling (or ballistic) current. Since we utilized a qPlus sensor, such a large current introduces significant electrical crosstalk that severely degrades the frequency shift signal, which is critical for our force spectroscopy. Thus, operating at $V_s = 0$ V was essential to suppress current-induced artifacts and isolate the purely mechanical mechanism.

However, we always explicitly measured the contact potential difference (CPD) for our tip-sample junctions. As noted in Section 11 of the Supplementary Information ("Contribution of electrostatic field"), the local CPD typically ranged between $|V_{\mathrm{CPD}}| \approx 0.2$ to 0.4 V depending on the specific tip apex condition. This implies a residual potential offset of up to 0.4 V during our $V_s = 0$ V measurements. We have already calculated the maximum possible electrostatic energy contribution under this maximum field, which yields an energy shift of only $\sim 0.045\ eV$. The electrostatic field effect is negligible, and our conclusion ($W < E_a$) remains robust.

Minor

- Page 2 line 68: Please add a verb after E_FS … the energies. I would use 'be'.

We thank the reviewer for their careful reading. We have corrected this typo by inserting the verb "be".

Reviewer #2 (Remarks to the Author):

The manuscript from Sugimoto and coworkers reports the inversion of a sumanene molecule on gold surface using qplus AFM and compares the mechanical work performed by the tip and the activation energy of the reaction. Although the experimental results are well performed, the explanation of the manipulation is not convincing, and maybe quite misleading. There has been quite a lot of representative papers in this area and the authors are recommended to cite those papers and discuss how the mechanism is different. After careful evaluation, I do not believe that the present manuscript meets the standard required for publication in Nature Communications, and I therefore recommend a major revision.

We thank Reviewer #2 for acknowledging that our experimental results are well-performed. We appreciate the opportunity to clarify our theoretical model. While single-molecule reactions triggered by thermal, optical, or electronic stimuli have been widely documented, purely force-driven reactions remain exceptionally rare. Our study offers a fundamentally new paradigm: showing that a mechanical reaction can proceed with a net energy input that is lower than the intrinsic activation barrier. Below we address your specific concerns.

My main concern is about how the authors connect the experimental results with W, which the authors define as the work done by the tip on the molecular system. First, the authors claims that W corresponds to the mechanical energy lost by the oscillator, but only Figure S7(c) shows the dissipation and the discussion on it is not enough.

As illustrated in our ideal conceptual framework (Fig. 1), the tip approaches the molecule from afar, triggers the inversion at the closest approach point, and retracts from the newly formed configuration. The mechanical energy lost by the cantilever over this single manipulation cycle corresponds to the area enclosed by the hysteresis loop between the approach and retraction force curves. We define this single-event net energy loss as W.

Experimentally, it is impossible to record a single continuous approach-retraction curve that captures the exact cycle of a single inversion event. Therefore, we reconstruct this trajectory by utilizing the stable force curve measured over a bowl-down molecule (representing the pre-jump approach path) and the stable force curve measured over a bowl-up molecule (representing the post-jump retraction path). We have rigorously discussed the validity of this approach in Section 8 of the Supplementary Information ("Validity of work calculation from frequency shift ").

Regarding the reviewer's point about energy dissipation, it is vital to distinguish between single-event energy loss and continuous cyclic dissipation. As is well established in NC-AFM dynamics, a one-time instantaneous structural transition does not manifest as the dissipation signal as explained in Section 8 of the Supplementary Information ("Validity of W calculation from frequency shift"). In contrast, the dissipation channel recorded by the AFM control loop represents an average over thousands of oscillation cycles while the tip continuously interacts with a molecule. As shown in Fig. S7C, the dissipation signal remains exceptionally small (~ 0.02 eV/cycle), which is more than an order of magnitude smaller than the work W being discussed. This explicitly validates our method of extracting W from the force curve hysteresis.

On the contrary, the authors attribute W to the hysteresis of the forward and backward. This is quite confusing. The force spectrum measures the force between tip and sample and the displacement is for tip, thus the integration is the work on tip. The hysteresis represents the different interaction on tip by the two different molecules (up/down) and this cannot be linked to the work on molecule.

We are deeply grateful to the reviewer for pointing out this critical error in our wording. Your description is entirely correct: W is technically the mechanical energy lost by the cantilever (i.e., the net work done *by the molecular system on the tip*). By the law of conservation of energy, this net mechanical energy lost by the cantilever over a complete cycle is transferred directly into the molecule-substrate system. A portion of this net input energy is stored as the increased internal energy

of the inverted molecule ($E_{\mathrm{FS}} - E_{\mathrm{IS}}$), and the remainder is dissipated into the substrate as heat (Q). Therefore, our mathematical formulation and the underlying physical conclusions remain fully intact. To resolve this point and ensure strict physical accuracy, we have thoroughly revised the descriptions throughout the manuscript:

Main Text (Page 3, Line 81):

Let W be the net energy input to the molecular system that interacts with the tip in this process.

Main Text (Page 5, Line 175):

This gain partially compensates for the energy consumed by the repulsive deformation, effectively reducing the net energy cost.

Main Text (Page 5, Line 192):

Force spectroscopy revealed that the net mechanical energy cost was significantly lower than the activation energy of the reaction.

Main Text (Page 5, Line 196):

..., enabling the reaction to proceed with sub-barrier energy consumption.

To clarify our concept, let us consider a macroscopic analogy of a cart crossing over a hill, which represents the activation energy barrier. To reach the top of the hill, the cart temporarily requires an input of energy at least equivalent to the barrier height. However, if we can efficiently recover energy as the cart rolls down the other side of the hill—akin to regenerative braking in macroscopic classical mechanics—the net mechanical work externally performed on the system over the entire cycle can be significantly lower than the actual height of the barrier. While this energy-recovery mechanism is a fundamental principle in macroscopic classical mechanics, our study demonstrates for the first time that this kind of physical logic can be reproduced at the single-molecule scale. In conventional single-molecule manipulations triggered by the injection of electrons or photons, energy is supplied in a one-way process; thus, the total energy consumption inevitably exceeds the activation barrier. In contrast, our mechanical approach using AFM achieves a single-molecule structural transformation with unprecedented net energy efficiency.

For another example, considering the force spectrum measured on two kinds of SnPc molecule on surface with Sn pointing up and down at the same z range, the difference between the two force spectra cannot be relative to the energy pushing a Sn atom through the molecule.

We appreciate the reviewer raising the interesting example of the SnPc molecule. If the tip approaches an SnPc molecule and triggers a conformational switch, two distinct force curves corresponding to the "up" and "down" configurations would indeed be obtained during the forward (approach) and backward (retraction) paths, respectively.

Our primary contention is that the area enclosed by this hysteresis loop represents the net mechanical energy injected into the molecular system (provided that the cyclic energy dissipation simultaneously acquired with the frequency shift is negligible, as demonstrated in our experiments).

When considering a single complete cycle of the cantilever oscillation—where the tip approaches the "up" molecule from a distance and then retracts far away from the newly inverted "down" molecule—the net mechanical energy lost by the cantilever is precisely what is transferred into the molecular system. Based on the first law of thermodynamics, this injected energy must be equivalent to the sum of the internal energy change of the SnPc/substrate system before and after the switching, and the unrecoverable thermal energy (heat dissipated into the substrate). Therefore, we believe our thermodynamic framework and the definition of W are physically rigorous and universally applicable to such force-induced molecular switching systems.

I recommend the authors to reconsider the theoretical model to explain the experimental finding based on the work from the guys like Ernst Meyer, Peter Grütter, or Alexander Schwarz. In my opinion, the authors can focus on the forward force spectrum alone and remove the background force (not on gold, but the hole in the assembling structure) to obtain the critical energy where the inversion of molecule happens, then they may get more convincing results.

We understand the reviewer's suggestion to focus solely on the forward (approach) force spectrum to isolate a critical force threshold, as is conventionally done. Indeed, focusing on the approach trace confirms our intuition that the reaction is driven by a localized Pauli repulsion pushing the center of the bowl.

However, we found that isolating a meaningful physical value from the absolute force threshold at the jump point is highly problematic. The force measured at the moment of inversion is a combination of the highly localized short-range Pauli repulsion (which drives the inversion) and the van der Waals (vdW) attraction exerted on the massive tip body. Because the vdW attraction depends strongly on the unpredictable, mesoscopic geometry of each specific tip apex, the absolute force value varies significantly from tip to tip, making the force value itself less physically informative.

Therefore, our study introduces a fundamentally different viewpoint: by focusing on the complete cyclic motion of the oscillating cantilever, we treat the AFM tip as a microscopic piston operating within a thermodynamic cycle. This thermodynamic analogy provides a new perspective in the field of NC-AFM, enabling us to elegantly demonstrate how vdW attraction can act as an energy-recovery mechanism.

To assist readers who are interested in the repulsive force threshold, we have compiled the vertical force thresholds measured at the moment of inversion and added a new histogram as **Fig. S10** in the Supplementary Information.

Besides the above issue, a more detailed discussion on Fig. 4 and Fig. S7 is also needed. If I understand it correctly, the Figure 4d shows the energy at different height on different structures, so the intersections should refer to tip height to make it easier for readers to understand. The intersections are points in Fig. 4c so it is hard to understand it easily.

We sincerely apologize for the formatting inconsistencies and drawing errors in Fig. 4 and Fig. S6, which caused unnecessary confusion. We have fully revised these figures to ensure strict color consistency and clarity. Specifically, we have added a new pane (**Fig. S6E**) that explicitly traces the total energy of the molecular system over time (following the sequential approach and retraction path from left to right) to make the trajectory intuitive for the reader.

Another question is that there should be 6 intersections between 7 structures, so the authors can show the rest to give the readers a full picture.

As the tip approaches, the system starts in the initial state (Structure 1), evolves through intermediate configurations 2 and 3, and reaches the transition state (Structure 4). At this exact instability point, the molecule abruptly snaps into the final inverted state (Structure 7). Therefore, the physical reaction pathway is strictly limited to the sequence 1 → 2 → 3 → 4 → 7. The remaining intermediate structures (5 and 6) pass by in an instant.
To give readers a full picture, we have added an explicit explanation to Section 6 of the Supplementary Information ("Theoretical validation of the energy saving mechanism"), referencing the newly added **Fig. S6E**:

Added text in Supplementary Information (Section 6):
We explicitly illustrate the time-evolution of the energy of molecular system in Fig. S6E. During the tip approach, the system sequentially transitions from the initial state (structure 1) through intermediate structures 2 and 3, eventually reaching the transition state (structure 4). At this exact moment, the molecule instantaneously snaps to the final state (structure 7). Therefore, the reaction pathway effectively follows the sequence 1 → 2 → 3 → 4 → 7. Note that the remaining structures (5 and 6) pass by in an instant.

Reviewer #3 (Remarks to the Author):

In this article, the authors revealed that the mechanical work W (measured by AFM) is smaller than

the activation energy Ea (obtained by NEB calculations), implying energy savings in the configuration switching of a bowl-shaped molecule. The authors have previously reported a similar work, i.e., repulsive-force-induced configuration switching of a single NO molecule on Cu(110) in Phys. Rev. Lett. 121, 116101 (2018). The idea is valuable in the context of mechanochemistry. However, several issues need to be carefully addressed, and I think the current version is below the requirements of Nat. Commun.

We sincerely thank Reviewer #3 for the valuable feedback and for recognizing that our research direction is highly valuable in the context of mechanochemistry. Many aspects of mechanochemical reactions remain poorly understood, and we conducted this study with the expectation that investigating these phenomena at the single-molecule level would significantly advance our fundamental understanding.

Previously, we reported the conformational change of a single NO molecule (from an upright to a flat orientation) induced by applying torque with an AFM tip [*Phys. Rev. Lett.* 121, 116101 (2018)]. In the present study, in addition to focusing on the transformation of the molecular structure itself, we place a paramount emphasis on the energy balance before and after the structural change. As discussed in detail later, in the previous NO system, the energy scale of the activation energy is relatively small, meaning that the contribution of the nuclear tunneling effect could not be completely ruled out. In contrast, the inversion of the sumanene molecule in this work involves a much larger activation energy scale, allowing us to safely neglect the tunneling effect (see Section 10 of the Supplementary Information, "Influence of tunneling effect in the molecular inversion"). Consequently, this system has provided an ideal and definitive platform to conclusively substantiate our energy-saving hypothesis ($W < E_a$).

Below, we address your specific questions regarding error margins, generality, and the experimental framework in a point-by-point manner.

1. The observed difference between W (~0.50 eV) and Ea (~0.67 eV) is about 0.17 eV. The authors should discuss whether this difference lies within the combined experimental and computational error margins. Specifically:

For W extraction from force curves: the choice of zero point, the correction for non-conservative forces, and the integration limits all introduce uncertainties.

Regarding the experimental W values, they were measured using seven different tips and exhibit a range from 0.3 eV to 0.5 eV, as shown in the histogram in Fig. 4A. We attribute this variation to differences in the tip apex geometry, which alters the contributions of the repulsive force driving the molecular inversion and the van der Waals (vdW) attractive force that enables the energy savings.

Crucially, even the maximum observed W value of 0.50 eV is lower than the calculated activation energy ($E_a = 0.67$ eV). Therefore, we believe that the condition $W < E_a$ is strongly demonstrated. Regarding the specific uncertainties raised by the reviewer:

Choice of zero point: The zero point of the force or frequency shift does not contribute to W because W is calculated from the hysteresis loop enclosed by the forward and backward paths.

Non-conservative forces: As discussed in Section 7 of the Supplementary Information ("Additional observables during inversion"), we have confirmed across all datasets that the energy dissipation signal is sufficiently small, introducing an uncertainty of only 10% at most.

Integration limits (cantilever oscillation amplitude): We perform precise amplitude calibration, and the error associated with the amplitude is at most 10%. We employ a highly accurate calibration method based on the displacement of the sample stage when changing the oscillation amplitude under tunneling current feedback control [*Y. Sugimoto, et al., Phys. Rev. B* 81, 245322 (2010)]. If "integration limits" instead refers to the mathematical integration error of the hysteresis loop area itself, this is a purely numerical calculation error and is negligible compared to the other sources of uncertainty.

Consequently, the variation in W is dominated by the condition of the tip apex, and our conclusion that $W < E_a$ remains solid.

Added text in Supplementary Information (Materials and Methods):

We employ a highly accurate calibration method of the oscillation amplitude based on the displacement of the sample stage when changing the oscillation amplitude under tunneling current feedback control, with an error of no more than 10 %.

For Ea from DFT: The authors should know the DFT can only treat ideal situations, at zero temperature and pressure. As W and Ea are respectively from experimental and computational approaches, is it scientifically correct to deduce the value of 0.17 eV as the energy savings? This is central, and clarification on this point is critical for the main findings of this work.

Our experiments are conducted at 5 K, which is sufficiently low compared to the energy scale of the potential landscape for the molecular structural inversion. Furthermore, the experiments are carried out under ultra-high vacuum (UHV) conditions, where the pressure is also sufficiently low.

Regarding the accuracy of the calculations, since this was also pointed out by Reviewer #1, we have added an explanatory text of Supplementary Information (Materials and Methods).

The energy saving of 0.17 eV represents the case where the theoretical and experimental values were closest. As indicated by the right arrow in Fig. 4A, the relationship $W < E_a$ holds true for all experimental trials, including this closest case.

As shown by the left arrow in Fig. 4A, W always exceeds the thermodynamic lower bound ($E_{\mathrm{FS}} - E_{\mathrm{IS}}$), which is also perfectly consistent. This lower bound corresponds to the limit where heat dissipation (Q) is zero, representing a scenario where the entire energy supplied by the tip is used exclusively to increase the internal energy of the molecule ($E_{\mathrm{FS}} - E_{\mathrm{IS}}$). Although this requires satisfying a highly specific energetic condition, it is theoretically permitted.

Revised Text in Main Text (Page 5, Line 168)

On the other hand, since $W > E_{FS} - E_{IS}$, $Q > 0$ *is satisfied, and the law of conservation of energy is not violated.*

2. In the authors' previous work (Phys. Rev. Lett. 2018, 121, 116101) and works from other groups, were a similar comparison between W and Ea performed? If so, was the W < Ea behavior already observed, or is it unique to the present molecular system (sumanene on Au(111))? Please clarify whether the energy-saving phenomenon is a general feature of repulsive-force-induced switching or specific to the bowl-shaped molecule.

We would like to emphasize that a comprehensive energy balance analysis comparing the energy input W and the activation energy (E_a) has never been conducted before. The paradigm of treating a single-molecule manipulation cycle as a thermodynamic engine cycle is an original concept introduced for the first time in this manuscript.

As derived in our "Theoretical Framework," the condition $W < E_a$ is a universal phenomenon that will occur in *any* force-driven reaction system, provided that a localized short-range repulsion coexists with a significant attractive interaction. It is a general feature of repulsive mechanochemical manipulation rather than a property unique to sumanene.

To date, there are only two other reported examples of purely repulsive-force-induced single-molecule reactions, both from our group: the conformational switching of a single NO molecule on Cu(110) [*Phys. Rev. Lett.* 121, 116101 (2018)] and the helicity switching of a graphene-nanoribbon (GNR)-fused helicene [*Chem. Sci.* 12, 13301 (2021)].

- In the GNR system, calculating a reliable E_a via DFT is nearly impossible because the molecule is part of an extended ribbon, meaning mechanical strain distributes widely and unpredictably.
- In the NO system, the mechanical work was roughly estimated to be 20–60 meV, which is lower than the calculated barrier of 125 meV. However, because the NO molecule is extremely light, the contribution of nuclear tunneling could not be definitively ruled out.

The sumanene molecule studied here provides an ideal testbed because its mass and energy scale are an order of magnitude larger, completely suppressing tunneling effects and allowing us to

unambiguously prove the mechanical energy-saving mechanism. To highlight this context, we have added a new section (**Section 12, "Studies in other molecular systems"**) to the Supplementary Information:

Added text in Supplementary Information (Section 12):

In our previous studies, we investigated repulsive-force-induced switching in other systems, such as the conformational change of a single NO molecule on Cu(110) [Phys. Rev. Lett. 121, 116101 (2018)] and the helicity switching of a graphene-nanoribbon (GNR)-fused helicene on Au(111) [Chem. Sci. 12, 13301 (2021)]. For the GNR system, quantifying the intrinsic activation energy (E_a) via DFT is exceptionally challenging because the molecule is part of an extended ribbon, meaning that mechanical strain and stress are distributed widely throughout the structure. For the NO system, the measured input energy was roughly estimated to be 20–60 meV, which is lower than the calculated barrier of 125 meV, hinting at a potential sub-barrier process. However, because the NO molecule is light, the contribution of nuclear tunneling could not be rigorously ruled out. In contrast, the sumanene molecule studied here possesses an energy scale and molecular mass an order of magnitude larger than those of NO, making nuclear tunneling completely negligible [see Section 10 of the Supplementary Information]. Thus, the sumanene/Au(111) system provides an ideal and definitive platform to experimentally and theoretically validate the $W < E_a$ hypothesis. This energy-saving mechanism is a general feature of force-driven reactions where short-range repulsion and middle-range attraction coexist, rather than an effect unique to sumanene. It is expected that similar analyses will be conducted on other molecular systems in the future.

3. The authors claimed that the contributions of tunneling and electrostatic are negligible. How do the authors assess the contribution of inelastic energy dissipation during the jump? This is particularly relevant because the hysteresis loop area includes both conservative and dissipative components.

The jump of the molecular inversion occurs instantaneously, on a timescale far faster than any other timescale, such as the cantilever oscillation. Because the inversion is completed at a speed faster than the travel speed of the tip, the energy generated at this exact moment cannot be recovered (as shown in the transition state [TS] in the middle panel of Fig. 1A). We define this unrecoverable energy as heat Q, which poses no issue within our thermodynamic theoretical framework. Please note again that we strictly consider the law of conservation of energy before and after the cycle ($W = E_{\mathrm{FS}} - E_{\mathrm{IS}} + Q$), as taught by the first law of thermodynamics.

The area of the hysteresis loop represents the mechanical energy lost by the cantilever during this cycle, which corresponds to the energy W injected into the molecular system. Since the area of the hysteresis loop is calculated using the force curves converted from the measured frequency shift, the

conservative forces are properly taken into account [see Section 8 of the Supplementary Information, ("Validity of work calculation from frequency shift")]. On the other hand, although the continuous energy dissipation is also observable in our measurements, we have evaluated its effect to be negligible [see Section 7 of the Supplementary Information ("Additional observables during inversion")].

4. The authors claimed that mechanical interactions allow a portion of the input energy to be recovered as work during the tip's motion. However, the tip is an oscillator; its mechanical energy change is governed by the driving force and dissipation. The potential energy landscape of the tip–molecule system may change before and after the inversion, resulting in different force–distance curves on approach and retraction. Consequently, the net mechanical energy loss of the cantilever is reduced.

We realize that our original phrase *"mechanical interactions allow a portion of the input energy to be recovered as work during the tip's motion"* caused confusion. We have modified the text in the revised manuscript to avoid any misunderstanding:
Main Text (Page 2, Line 62): *By comparing the net energy consumption derived from force spectroscopy with the activation energy obtained using density functional theory (DFT) calculations, we verified that the reaction proceeds with sub-barrier energy consumption.*
Main Text (Page 3, Line 97): *...mechanical interactions allow energy saving (detailed simulation provided in Supplementary Information).*

Regarding the dynamics of the tip as an oscillator: during a single steady-state oscillation cycle where a reaction occurs, we do not need to explicitly incorporate the external driving signal. In frequency-modulation AFM, the external drive energy is continuously adjusted to perfectly balance the intrinsic material damping of the cantilever during each cycle [F. J. Giessibl, *Rev. Mod. Phys.* 75, 949 (2003)]. For a short time after a jump occurs, the control system that maintains a constant amplitude does not function. The instantaneous reaction alters the interaction potential landscape between the approach and retraction. This alters the force trajectory, inducing the net mechanical energy loss (W) of the cantilever over that cycle. To make this easier to understand, the newly added Fig. S6E illustrates how the energy of the molecular system changes over time as the tip approaches and retracts.

5. The discussion does not relate the central finding $W < Ea$ to any established theories of reactions (e.g., Bell model, Kramers' rate theory). Even though reference [47] cites the Kramers review, it is not discussed in the text. How does the present result fit into or differ from the classical framework where an applied force lowers the effective barrier but still requires an energy input at least equal to the remaining barrier height?

We appreciate the opportunity to clarify how our work connects to and differs from standard mechanochemical models. In conventional frameworks operating at finite temperatures (such as the standard Bell model or Kramers' rate theory), an applied mechanical force acts to lower the activation energy, and the system eventually overcomes the remaining, reduced barrier via thermal fluctuations ($k_B T$).

However, a critical distinguishing feature of our experiment is that it is conducted at a cryogenic temperature of 5 K. At 5 K, thermal fluctuations are completely negligible ($k_B T \approx 0.4$ meV), meaning the system possesses zero thermal energy to overcome a remaining barrier. Therefore, to drive a structural transformation at 5 K, the effective activation barrier must be compressed completely to zero by the mechanical force of the approaching tip. The structural inversion occurs exactly at the mechanical instability point (the crossover point iii in Fig. 4D) where the barrier vanishes entirely.

By emphasizing this "vanishing barrier" mechanism at 5 K, our finding does not contradict classical rate theories; rather, it demonstrates the absolute low-temperature limit of force-driven processes. It reveals that by taking advantage of mechanical energy recovery via long-range interactions, a single-molecule reaction can be completed with ultimate net energy efficiency.

Added text in Supplementary Information (Section 6):

Finally, we discuss the relationship between our findings and established mechanochemical frameworks, such as the Bell model and Kramers' rate theory. In conventional mechanochemical models operating at finite temperatures, an applied mechanical force acts to lower the activation energy barrier, and the system subsequently overcomes the remaining, reduced barrier via thermal fluctuations ($k_B T$). In sharp contrast, our experiments are conducted at a cryogenic temperature of 5 K, where thermal fluctuations are entirely negligible ($k_B T \approx 0.4$ meV). Under these low-temperature conditions, the system possesses insufficient thermal energy to clear any remaining barrier. Therefore, to drive the structural transformation at 5 K, the effective activation barrier must be compressed completely to zero by the mechanical force of the approaching tip. The structural inversion thus takes place precisely at the mechanical instability point, where the energy barrier vanishes entirely (corresponding to the crossover point iii in Fig. 4D). This "vanishing barrier" mechanism does not contradict classical rate theories; rather, it delineates the absolute low-temperature limit of force-driven processes.

6. In the beginning of the results section, the STM image (Fig. 2B) labels two distinct features as U and D, but to a reader these merely appear as different bright spots. The authors should first schematically explain the two molecular orientations (bowl-up vs. bowl-down) in Fig. 2A, and then use high-resolution AFM images and DFT calculations to unambiguously assign the structures.

We thank the reviewer for this constructive editorial suggestion. We have adjusted the positions of the U and D labels in Fig. 2 to make them clearer. We have also explicitly modified the introductory sentence to guide the reader:

Revised text in Main Text (Page 3, Line 102):

...because its steric structure, "bowl-down" (D) and "bowl-up" (U), allows for inversion via repulsion (Fig. 2A).

7. The manipulation experiments were performed exclusively on the D (bowl-down) molecules. After converting D to U, can the reversed process be achieved (e.g., by pulling the tip or using a different approach)? Can the U (bowl-up) molecules also be inverted to the D configuration by applying repulsive force? Demonstrating reversibility would greatly strengthen the claim of controlled mechanical switching. Please discuss whether such experiments were attempted and, if not, state the limitations.

We agree that demonstrating reversibility is an excellent target for molecular switches. We systematically attempted to induce the reverse transition [from bowl-up (U) back to bowl-down (D)] by approaching the tip directly over the center of a U-shaped molecule. However, no reverse inversion was ever observed. To snap a bowl-up molecule back down, one would need to locally pull the central ring upward. The attractive force exerted by our tip apex was either insufficient in magnitude or lacked the sub-nanometer spatial confinement needed to pull the center selectively, relative to the short-range repulsion.

Crucially, the absence of this reverse switching is a mandatory prerequisite for our experimental framework. During the retraction phase after a successful D-to-U inversion, the tip must pass back through the attractive interaction regime. If the attractive force were strong enough to pull the molecule back into a D configuration, the net inverted state would immediately undo itself, and a stable inversion could never be achieved or observed.

We also attempted to apply repulsive forces to the outer rim of the U-molecules to force a down-switching, but this triggered complex, asymmetric azimuthal molecular rotations rather than a clean inversion. This confirms that applying a highly localized repulsive force exactly at the high-symmetry center of the buckybowl is essential to cleanly drive the inversion. We have added this detailed discussion as **Section 13 ("Trial of reverse molecular inversion")** in the Supplementary Information.

Added text in Supplementary Information (Section 13):

We systematically attempted to induce the reverse transition [from bowl-up (U) to bowl-down (D)] by approaching the tip to the center of a U-shaped sumanene molecule. However, no reverse inversion was observed. To trigger the reverse process, one would need to locally pull the center of the bowl

upward, but the attractive force exerted by the tip was either insufficient in magnitude or lacked the necessary spatial confinement compared to the short-range repulsion. Notably, the absence of this reverse switching is a critical prerequisite for our experimental validation; during tip retraction after a successful D-to-U inversion, the tip inevitably traverses the attractive interaction regime. If the attractive force were strong enough to pull the molecule back into the D configuration, the net inversion could never be stabilized or observed. We also attempted to apply repulsive force to the outer rim of the U molecules to see if it could force a down-switching, but this resulted in complex, non-symmetric molecular rotations rather than a clean inversion. This highlights that applying a highly localized repulsive force exactly at the high-symmetry center of the molecule is essential to cleanly drive the structural transformation.

8. The main text lists references up to [36]. References [37]–[48] should appear in the supplementary materials but are not clearly marked. Please indicate which references belong to the main text and which to the SI, or renumber them accordingly to avoid confusion.

We apologize for the confusion caused by the overlapping citation numbers. We have fully separated and renumbered the reference lists so that the Main Text and the Supplementary Information references are independent and clearly marked.

Response to the Editor and Reviewers

Thank you very much for reviewing our manuscript entitled "Single-molecule structural transformation by mechanical force" and for providing us with the valuable opportunity to submit a revised version. We also sincerely thank the three reviewers for their time, constructive feedback, and deep insights.

We would like to express our gratitude to Reviewers 1 and 3 for supporting the publication of our work. We also highly appreciate Reviewer 2 for providing further insightful comments to improve the manuscript. We have carefully addressed all the concerns raised by Reviewer 2.

Because this study takes a fundamentally different approach to analyzing AFM-based atomic and molecular manipulations compared to conventional methods, we intend to opt for the transparent peer review process to make this Response Letter publicly available upon acceptance. We believe that the detailed explanations provided herein will serve as a valuable reference and clarify our methodology for researchers who are well-acquainted with conventional frameworks. We hope that the revised manuscript is now fully suitable for publication in *Nature Communications*.

Reviewer #1 (Remarks to the Author):

In their rebuttal, Iwata et al. properly addressed my concerns and seem to clarify the concerns of the other reviewers. I agree with the authors that showing that a mechanochemical reaction can happen despite having a larger activation energy barrier is quite interesting. To me, the manuscript is ready to be published.

We are deeply grateful to Reviewer 1 for the positive evaluation and for supporting the publication of our manuscript. We are particularly encouraged by the reviewer's concurrence on the core significance of our findings—namely, that a mechanochemical reaction can indeed occur even under the $W < E_a$ condition. This endorsement is highly meaningful to us, and we hope it helps the Editor and the other reviewers recognize the validity and impact of our non-conventional thermodynamic approach.

Reviewer #3 (Remarks to the Author):

In the revised version of manuscript, I see that the authors have addressed all the concerns and provided convincing responses. I therefore recommend acceptance of it for publication in Nature Communications.

We sincerely thank Reviewer 3 for reviewing our revised manuscript and for recommending its acceptance for publication in Nature Communications. We are very pleased that our previous responses were found convincing and that the clarifications successfully addressed all of the reviewer's concerns. We believe this strong support further reinforces the readiness of our manuscript for publication.

Reviewer #2 (Remarks to the Author):

I have read the reply to me, and I think that the revised manuscript only solves minor issues. Although the experiments were well-performed and the presentation has been improved, the explanation on the theoretical model is still not convincing. Therefore, it is still below the requirements of Nat. Commun. in this version.

We sincerely thank Reviewer 2 for the continued evaluation of our manuscript. We appreciate the acknowledgment that our experiments were well-performed and that the presentation has been improved. Regarding the theoretical model, we have provided deeper explanations and additional simulations below to fully address the reviewer's remaining concerns.

First, there are still some minor issues. The tip used in all images should be denoted to show which kind of tip is used. I didn't find any experimental results which confirm the reversion by a metal tip, while there are only theoretical results in the SI. If the molecules cannot be inversed by metal tip experimentally, what was the analyzation for? In addition, the authors claim that the tip is sumanene-terminated, why would an Au tip be used in Fig. S6 for energy analyzation?

We appreciate this important comment, which has allowed us to significantly clarify the descriptions in our manuscript.
In scanning probe microscopy, it is generally difficult to determine the exact atomic configuration of the tip apex with absolute certainty. To maintain rigorous scientific accuracy, we have explicitly specified the tip state in the figure captions only when we intentionally prepared a CO-terminated tip. When molecular orbitals are clearly emphasized (as seen in the STM images of Fig. 3 and Fig. S4), the tip is widely considered to be a molecule-modified tip. However, we prefer to avoid making definitive claims about the tip apex state in the caption to maintain scientific accuracy. Conversely, proving that a tip is purely metallic is even more challenging. While highly symmetric STM images like those in Fig. S1 and Fig. S9 suggest a metallic tip, the possibility of a molecule-terminated tip cannot be entirely ruled out.
Regarding Fig. S4, after performing the inversion experiments shown in the STM images (A and B),

we intentionally prepared a CO-terminated tip to acquire the AFM image (C). We have added this explanation to the caption of Fig. S4 as follows:

Added text in Caption for Fig. S4:

(C) AFM image of the same area acquired with a CO-terminated tip.

We believe that whether the tip apex is metallic or molecule-terminated is not the governing factor in the molecular inversion process. We have experimentally confirmed that the inversion of the sumanene molecule is driven by the **repulsive force** from the tip. Furthermore, our DFT calculations in Fig. S5 successfully reproduced the repulsion-driven inversion for both molecule-terminated and bare metal tips. This Pauli repulsion acts universally, regardless of the chemical state of the tip apex. This is in stark contrast to attraction-driven manipulations, where the processability is strongly influenced by whether the tip is a highly reactive bare metal or an inert molecule-terminated tip [22, 23].

In the previous version, a bare metal tip model was used in Fig. 4 and Fig. S6 simply as a straightforward prototype to demonstrate that the $W < E_a$ condition can be satisfied. To address your concern and make the theoretical analysis more comprehensive, **we have newly performed simulations using a sumanene-capped Au tip model and added the results as Fig. S11.** Notably, the sumanene-capped tip model also successfully reproduced the $W < E_a$ condition. We have updated the text and added the new figure to the Supporting Information.

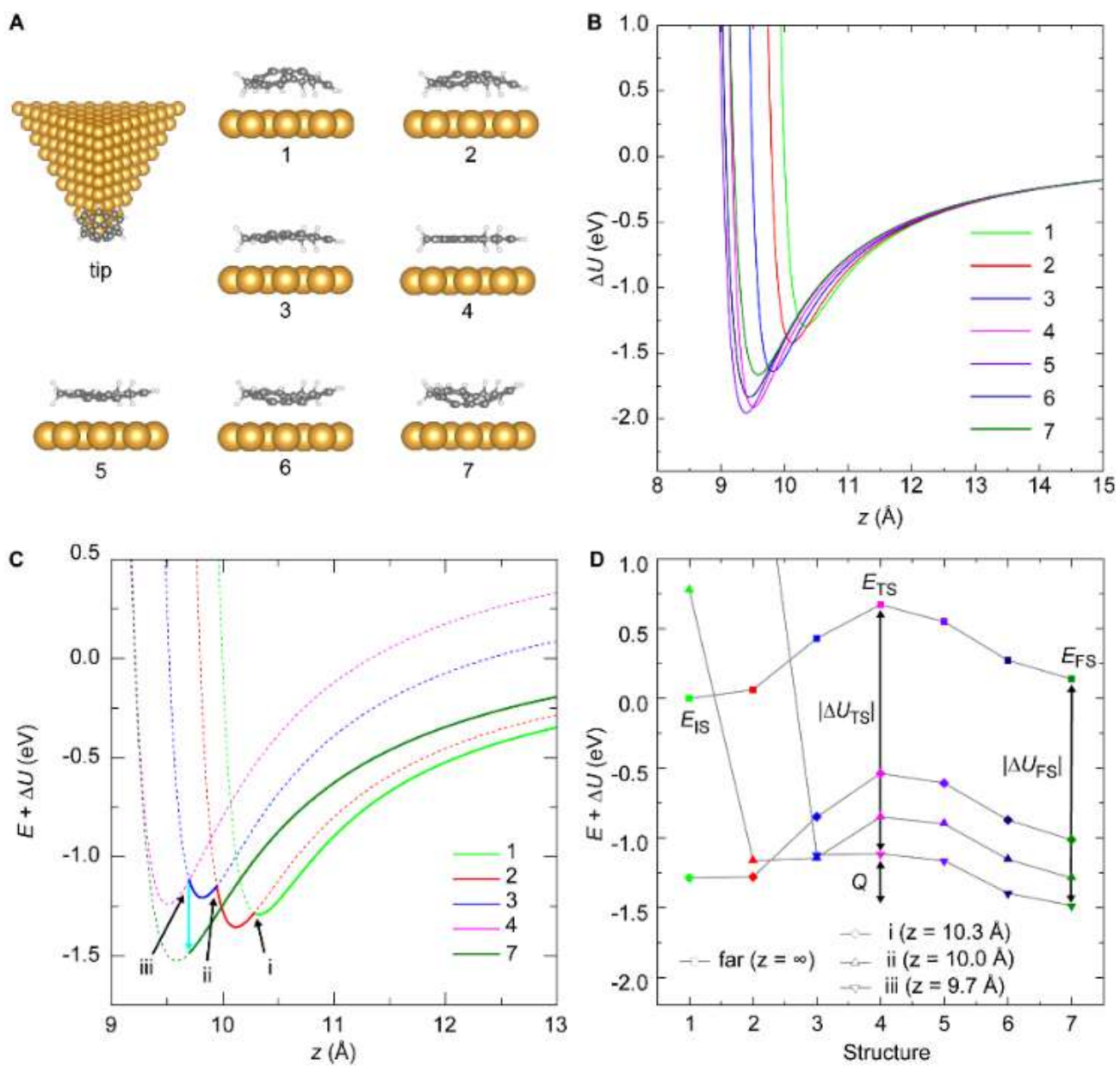

Fig. S11. Energetics of the inversion. (A) Geometries of the sumanene-terminated Au tip (the truncated tetrahedral Au cluster composed of 219 atoms) model and the sumanene molecule in the molecular layer on Au(111) along the reaction coordinate for the inversion obtained using the NEB

method. Structures 1, 4, and 7 represent the initial, transition, and final states, respectively, while structures 2, 3, 5, and 6 are intermediate states. Only the inverted sumanene molecule and Au atoms in the first layer are shown. (B) Lennard-Jones potential (ΔU) between the molecular structures and the tip model shown in (A). (C) Total energy curves obtained by adding the Lennard-Jones potential shown in (B) to the intrinsic NEB energies. Intersections i, ii, and iii correspond to transitions between structures 1–2, 2–3, and 3–4, respectively. (D) Evolution of energy profile along the reaction coordinate. ΔU_{TS}, ΔU_{FS}, and Q are indicated by double-headed arrows. This model yields $\Delta U_{\mathrm{TS}} < \Delta U_{\mathrm{FS}}$ (ΔU_{TS} =-1.79 eV vs. ΔU_{FS} =-1.62 eV), successfully reproducing the $W < E_{\mathrm{a}}$ condition.

Added text in Main Text (The paragraph immediately preceding the Conclusions chapter):
The Au tip capped with the sumanene also reproduced the $W < E_a$ condition although the value of W differed slightly (Fig. S11). The variance in measured W (Fig. 4A) likely reflects the geometric diversity of the tips.

The schematic in Fig. 1A is not suitable because the three tip positions are quite misleading that they look like in the same oscillation period. If I understand it correctly, they are not in the same oscillation period and they only represent "far-medium-close". Otherwise, the discussion on dissipation should be provided. The schematic may need to be improved.

We apologize for the ambiguity, but we would like to clarify that **Figure 1A does indeed represent a single cycle of the cantilever oscillation**. The chronological progression of the three panels corresponds precisely to one oscillation period ($1/f$). To prevent any further misunderstanding, we have explicitly stated this in the caption of Fig. 1.
The molecular inversion is expected to occur within a single swing of the cantilever, and this dynamic process is what we have schematically illustrated. Regarding energy dissipation, it is comprehensively discussed in Sections 7 and 8 of the Supporting Information. During this single oscillation cycle, the cantilever loses an amount of energy equal to W. Under constant-amplitude control, this energy deficit is dynamically compensated for by the external excitation circuit (automatic gain control, AGC) in subsequent cycles.

Added text in Caption for Fig. 1:
(A) Schematic illustration of potential energy landscapes during the reaction over one cycle of cantilever oscillation.

By the way, the Fig 2A looks like the molecule can be reversed by a metal tip, which may also be modified or the author should show the relative experimental results at least.

Thank you for this constructive suggestion. Since Figure 2 is not intended to illustrate the inversion operation itself, we have removed the tip from Fig. 2A. It now serves strictly as a schematic defining the "bowl-down" (D) and "bowl-up" (U) configurations. This modification makes the logical flow of the figure much clearer. Thank you for your helpful advice.

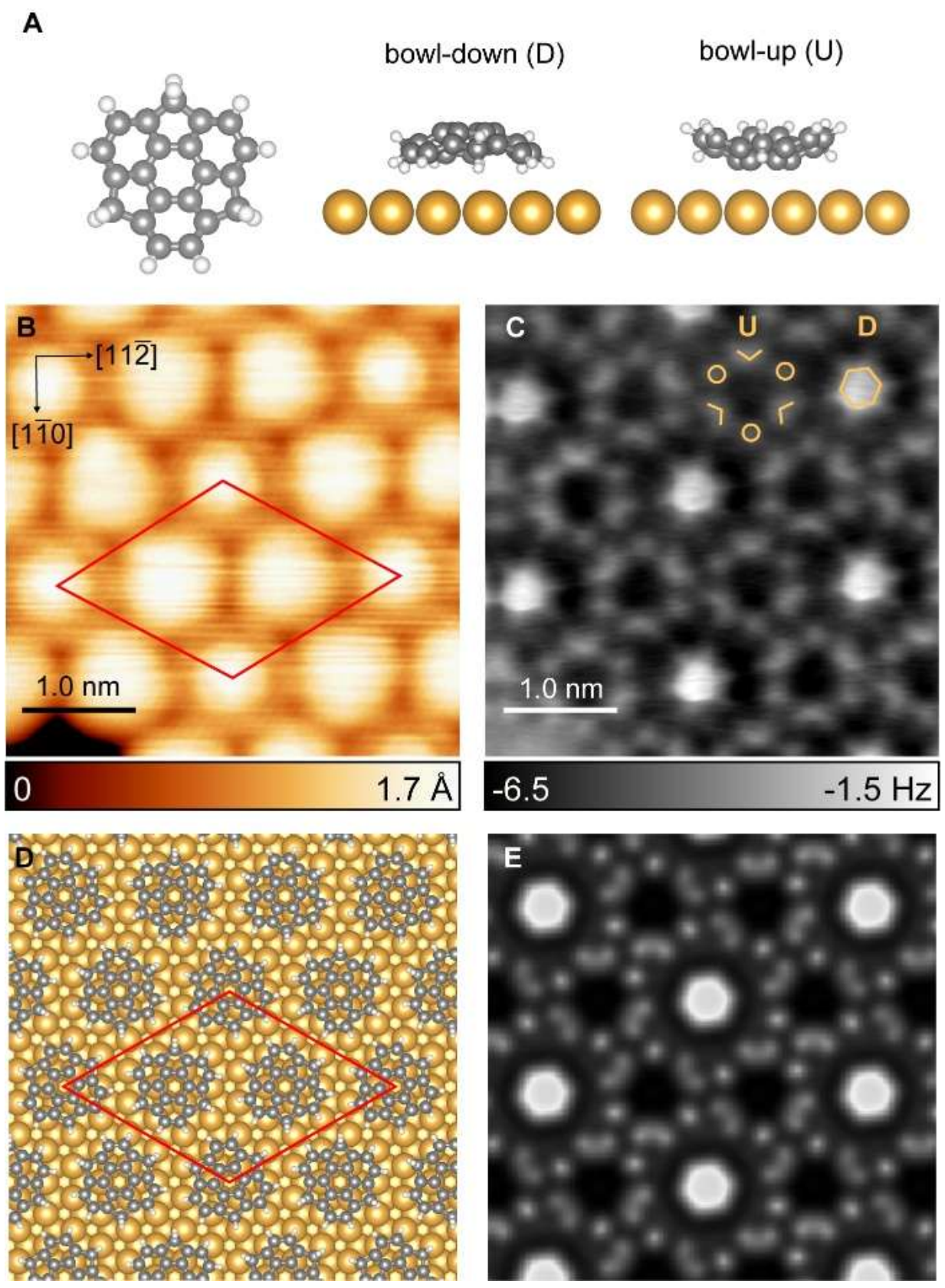

Fig. 2. Structure and imaging of sumanene on Au(111). (**A**) Top and side views of the sumanene molecule. (**B**) STM image of a self-assembled sumanene monolayer on Au(111) acquired with a CO-terminated tip (V_S = 50 mV, I_t = 10 pA). The red rhombus indicates the unit cell. (**C**) Constant-height AFM image of the same area as in (B). Two distinct molecular appearances are denoted as U (bowl-up) and D (bowl-down). Orange lines schematically highlight characteristic features. (**D**) Energetically most stable adsorption structure obtained by DFT calculations. Gray, white, and yellow spheres represent carbon, hydrogen, and gold atoms, respectively. (**E**) Simulated AFM image corresponding to the structure in (D) using the probe particle model.

The above issues are solvable. The main issue is the theoretical model and in the following I have to explain my main concern.
I still have questions on the comparison between W and Ea, which is the central find of this manuscript. I also agree that the potential energy landscape of the tip–molecule system changes before and after the inversion. The comparison between the forward and backward energy spectra is not related to the energy barrier of the inversion.
The authors claim that at the lowest tip position the inversion emerges. Therefore, the interaction force and energy between tip and molecule at that position is quite important to evaluate the barrier of the inversion. I understand that this point of view is "conventional" for the authors but it has been correct in most cases and well-accepted in the community. The effort on removing the background force is a known choice to get quantitative analyzation on the barrier, which is what I recommended in the first time.

We completely agree with the reviewer that the forward force and potential energy curves up to the point of inversion are crucial for understanding atomic and molecular manipulations. The values of the force and potential energy at the exact moment of the "jump" must inherently correlate with the reaction barrier.
In fact, following the reviewer's suggestion in the previous revision, we included a histogram of the forces measured at the exact moment of inversion in Fig. S10. This data clearly demonstrates that the inversion is driven by a short-range repulsive interaction. However, the values of the repulsive force at the transition point exhibit significant scattering, making a direct, quantitative correlation with the activation barrier highly challenging.
This scattering arises because the measured total short-range force is a superposition of the driving repulsive force and the non-driving attractive interactions. While the repulsive force is determined primarily by the point of contact between the tip and the molecule, the attractive force is highly sensitive to the structure of the tip because it involves contributions from multiple atoms at the tip. Because the atomic arrangement of the tip changes from experiment to experiment, the total force and potential energy at the moment of manipulation inevitably scatter (which is the phenomenon we aimed to explain in our previous response).
Regarding the background force removal, we estimated the background contribution in this study by acquiring force curves on the bare Au(111) terrace adjacent to the target sumanene molecule. By subtracting this background from the force curves measured over the molecule, we extracted and analyzed the short-range forces (see Fig. S7).
We fully understand the reviewer's ideal recommendation: to measure the background force directly

over a molecular vacancy created by extracting the molecule after the inversion experiment. To execute this, one must perform the inversion, remove the molecule to create a vacancy, and then record the background force curve—all while perfectly maintaining the microscopic state of the tip apex. This sequence represents an extraordinarily challenging experiment in SPM, which we must relegate to future work. Combining such ideal measurements with the thermodynamic insights established in this paper will undoubtedly yield deeper insights in the future.

As members of this research community, we deeply respect and recognize the immense value of what we termed the "conventional method." Our group has been actively engaged in AFM-based atomic and molecular manipulation for over two decades. Regarding recent molecular manipulations closely related to this work, we have already included discussions on NO molecular conformational changes and GNR switching in Section 12 of the SI ("Studies in other molecular systems").

Furthermore, we have pioneered numerous studies combining atomic manipulation with precise force spectroscopy [R1–R7], measuring the interaction energies at the exact moment of manipulation. Through these experiences, we have learned that while interaction energies and activation barriers are correlated, mapping one to the other quantitatively is far from trivial. For instance, there are cases where a certain tip yields a potential energy value that reliably triggers atom manipulation, but when that same value is achieved with a different tip, the atomic manipulation fails [R6]. Therefore, experimentally determining the exact value of an activation barrier remains a formidable challenge.

Our current manuscript introduces a complementary paradigm: analyzing the hysteresis loop through a thermodynamic framework to establish a rigorous boundary condition ($W < E_a$) for the activation barrier. We firmly believe that validating this method across various systems will establish it as a powerful, alternative tool for the community.

[R1] 'Mechanism for room-temperature single-atom lateral manipulations on semiconductors using dynamic force microscopy'
Y. Sugimoto, P. Jelinek, P. Pou, M. Abe, S. Morita, R. Perez, and O. Custance
Physical Review Letters vol. 98 (2007) pp. 106104 1-4

[R2] 'Complex patterning by vertical interchange atom manipulation using atomic force microscopy'
Y. Sugimoto, P. Pou, O. Custance, P. Jelinek, M. Abe, R. Perez, and S. Morita
Science vol. 322 (2008) pp. 413-417

[R3] 'Role of Tip Chemical Reactivity on Atom Manipulation Process in Dynamic Force Microscopy'
Y. Sugimoto, A. Yurtsever, M. Abe, S. Morita, M. Ondracek, P. Pou, R. Perez, and P. Jelinek
ACS Nano vol. 7 (2013) pp. 7370-7376

[R4] 'Mechanical gate control for atom-by-atom cluster assembly with scanning probe microscopy'
Y. Sugimoto, A. Yurtsever, N. Hirayama, M. Abe, and S. Morita

Nature Communications vol. 5 (2014) pp. 4360 1-7

[R5] ‘Interplay between switching driven by the tunneling current and atomic force of a bistable four-atom Si quantum dot’

S. Yamazaki, K. Maeda, Y. Sugimoto, M. Abe, V. Zobac, P. Pou, L. Rodrigo, P. Mutombo, R. Perez, P. Jelinek, and S. Morita

Nano Letters vol. 15 (2015) pp. 4356-4363

[R6] ‘Role of lateral forces on atom manipulation process on Si(111)-(7x7) surface in dynamic force microscopy’

A. Yurtsever, M. Abe, S. Morita, and Y. Sugimoto

Physical Review B vol. 96 (2017) pp. 155412 1-8

[R7] ‘First-principles study of lateral atom manipulation assisted by structural relaxation of a scanning tip apex’

B. Enkhtaivan, Y. Sugimoto, and A. Oshiyama

Physical Review B vol. 96 (2017) pp. 155417 1-7

The last issue is about the energy conservation. There are two kinds of energy conservation at least. The first is the oscillation of the tip. If there is any dissipative interaction between tip and sample, it requires more energy to keep the oscillation of the tip. This has been proven by the authors that the dissipative interaction is negligible. In this case, the second part is more important. When the tip approaches to sample, the potential energy between tip and sample is changing but the kinetic energy of the tip or sample is unchanged. Where does the energy go or come from? It is the tip holder (if it is the tip which moves, otherwise this will be the sample holder). The interaction between tip and sample does the positive energy and the tip holder does the negative energy, or vice versa. Thus, the difference between the two spectra (W) has nothing to do with the energy transfer to the molecule. The authors should not use the conservation of energy in the response to my concern about the work on molecule.

We appreciate the reviewer’s rigorous breakdown of energy conservation. We would like to address the two components—cantilever oscillation energy and tip kinetic energy—separately to clarify our thermodynamic framework.

First, regarding the steady-state oscillation energy of the cantilever: As discussed in Section 7 of the SI, the energy dissipation measured during a normal cycle (excluding the exact frame where control is momentarily lost during the jump) is extremely small, remaining below 0.02 eV/cycle (see Fig. S7). This indicates that while the cantilever loses a tiny fraction of energy each cycle due to non-conservative interactions with the sample, this loss is continuously replenished by the automatic gain control (AGC) circuit. Because this dissipation is a continuous cyclic process and its magnitude (0.02 eV) is negligible compared to the energy scale of the molecular inversion, it does not affect the single-

event energetics.
However, at the exact cycle where the inversion occurs, the cantilever abruptly loses an amount of energy equal to W. This single-event energy drop slightly reduces the oscillation amplitude during that specific swing, and the AGC circuit subsequently does work to restore the amplitude in the following cycles. Because this is a one-time discrete event embedded in a continuous measurement, it is too transient to be registered as a conventional, time-averaged dissipation value [eV/cycle] (as detailed in SI Section 8).

Second, regarding the kinetic energy of the tip and the role of the holders: In our experimental setup, the cantilever holder is magnetically clamped to a piezoelectric actuator at the top of the tube scanner, while the sample stage remains stationary. Our force spectroscopy records the frequency shift of the oscillating cantilever as a function of the quasi-static displacement of the cantilever holder controlled by the scanner.
When the molecular inversion occurs, it takes place at the point of closest approach within a single cantilever swing. As the tip swings downward toward the sample, the potential energy of the tip-sample system decreases due to attractive van der Waals interactions, and **this potential energy is directly converted into the kinetic energy of the tip** (as detailed in the section "Mechanism of Energy Savings" in the main text). This dynamically acquired kinetic energy is precisely what enables the system to overcome the structural deformation under the $W < E_a$ condition.
Crucially, because the mechanical displacement of the cantilever holder by the piezo scanner occurs at a quasi-static speed relative to the high-frequency oscillation of the cantilever (18.5 kHz), the piezo drive system does no mechanical work during a single oscillation period (since work is the integral of force over displacement, and holder displacement during one swing is virtually zero). Therefore, no energy is transferred to or from the tip holder during the inversion event itself.

Based on this clear decoupling, the total energy of the oscillating cantilever can be considered within that specific cycle, allowing us to implement a rigorous thermodynamic approach. The difference between the forward and backward spectra (W) reflects the net mechanical work lost by the cantilever, which must enter the molecule-surface system. This justifies our application of energy conservation to evaluate the bounds of the work done on the molecule.
We note that non-oscillating STM manipulation is also capable of inducing molecular inversion; however, in that case, the energy balance must indeed incorporate the electrical energy supplied to the piezoceramics, as discussed in Section 8 of the SI.
